# Evaluating AI-Generated Molecules for Drug Discovery: From Generic Metrics to Translational Readiness

**DOI:** 10.3390/ijms27135916

**Published:** 2026-06-30

**Authors:** Xiaomeng Liu, Huanxiang Liu

**Affiliations:** Centre for Artificial Intelligence Driven Drug Discovery, Faculty of Applied Sciences, Macao Polytechnic University, Rua de Luís Gonzaga Gomes, Macau SAR, China

**Keywords:** de novo drug design, deep generative models, AI-generated molecules, evaluation metrics, medicinal chemistry, target-aware evaluation, structure-based assessment, translational readiness

## Abstract

Artificial intelligence-driven molecular generation has become an increasingly used computational approach for proposing candidate chemical structures in early-stage drug discovery, yet the practical value of the molecules produced is often difficult to judge. Many studies still rely mainly on model-level metrics such as validity, uniqueness, novelty, and diversity. These metrics describe whether a generator produces parsable, non-redundant structures that extend beyond a reference set, but they do not show whether the molecules are chemically credible, biologically relevant, or experimentally actionable. AI-generated molecules are best treated as testable hypotheses requiring staged, complementary evidence rather than judgments based on generic generative statistics. We discuss the interpretive limits of common metrics, examine complementary levels of evaluation including medicinal chemistry feasibility, target relevance and prediction reliability, structure-based plausibility, and translational readiness, and identify recurring failure modes such as false novelty, reward exploitation, predictor bias, docking overinterpretation, and selective reporting. We propose a six-stage, failure-aware evaluation framework spanning molecular correctness, medicinal chemistry feasibility, novelty and diversity in context, target relevance and prediction reliability, structure-based plausibility, and translational readiness. This framework does not replace experimental validation; instead, it helps align computational claims with the strength of supporting evidence and promotes more transparent and reproducible evaluation of AI-generated molecules in drug discovery.

## 1. Introduction

Artificial intelligence (AI)-driven molecular generation is now widely used to propose new chemical structures in early-stage drug discovery. Rather than selecting compounds only from pre-enumerated libraries, generative models learn patterns from existing chemical data and generate molecular structures under predefined design objectives [1,2,3]. These objectives may include reproducing a reference chemical distribution, optimizing physicochemical properties, designing analogues of known compounds, or enriching molecules predicted to be relevant to a biological target [1,2,3,4]. In this context, molecular generation is best understood as a hypothesis-generating approach. It can broaden the pool of molecules considered for further study, but it does not, by itself, establish that the generated molecules are useful candidates for drug discovery.

The rapid progress of molecular generative models has increased the need for appropriate evaluation standards. Commonly reported metrics, shaped in part by benchmarks such as GuacaMol and MOSES, include validity, uniqueness, novelty, and diversity [1,2]. These measures capture basic aspects of model behavior, such as whether generated structures are chemically parsable, non-redundant, distinct from the training set, and distributed across chemical space. However, their scope is limited. They do not establish whether a generated molecule is chemically feasible, biologically relevant, structurally plausible, or suitable for experimental testing.

This distinction has practical consequences in drug discovery. A molecule may be valid yet synthetically unrealistic, novel yet closely related to known chemistry, diverse yet unrelated to the intended target, or highly ranked by a predictive model despite lying outside its reliable domain. Drug-likeness rules, quantitative estimate of drug-likeness (QED), synthetic accessibility (SA) scores, scaffold-based analysis, and structural-alert filters provide useful early checks, although each addresses only part of the evaluation problem [5,6,7,8,9,10]. Specifically, Lipinski’s rules and QED summarize property-based drug-likeness instead of target activity or safety [6,7]. Meanwhile, synthetic accessibility scores provide approximate estimates of chemical tractability [8]. As for pan-assay interference compounds (PAINS) and other structural-alert filters, they should be interpreted in relation to the relevant chemical series and assay system rather than applied as automatic exclusion criteria [10].

Target-directed molecular generation introduces additional complexity. On the one hand, activity predictors, quantitative structure–activity relationship (QSAR) models, drug–target interaction (DTI) models, and drug–target affinity (DTA) models are often used to evaluate or guide generated molecules [4,5]. On the other hand, their predictions depend on multi-scale considerations, involving the quality of training data, endpoint definition, validation strategy, and applicability domain [5]. This issue becomes more pronounced when generated molecules are structurally distant from known compounds. In this setting, a high predicted score should be treated as a computational hypothesis, not as evidence of experimental activity. The risk is greater when the same surrogate model is used as the optimization reward, because the generator may learn to increase the predicted score without producing molecules with greater medicinal chemistry value [3].

Structure-based assessment can provide additional evidence when a target structure or a reliable structural model is available. Docking, pose inspection, interaction analysis, rescoring, molecular mechanics/generalized Born surface area (MM/GBSA), and molecular dynamics (MD) simulations can help assess whether generated molecules fit a binding site and whether the proposed interactions are chemically interpretable [11,12,13]. Docking scores and short MD stability analyses are mainly useful for prioritization and structural-plausibility assessment, whereas MM/GBSA and other MD-based free-energy calculations can support binding-affinity-oriented prioritization when properly applied. In silico tools to predict absorption, distribution, metabolism, excretion, and toxicity (ADMET) can also identify early developability risks, but they do not replace experimental pharmacokinetic or safety studies [14,15].

**Figure 1:**
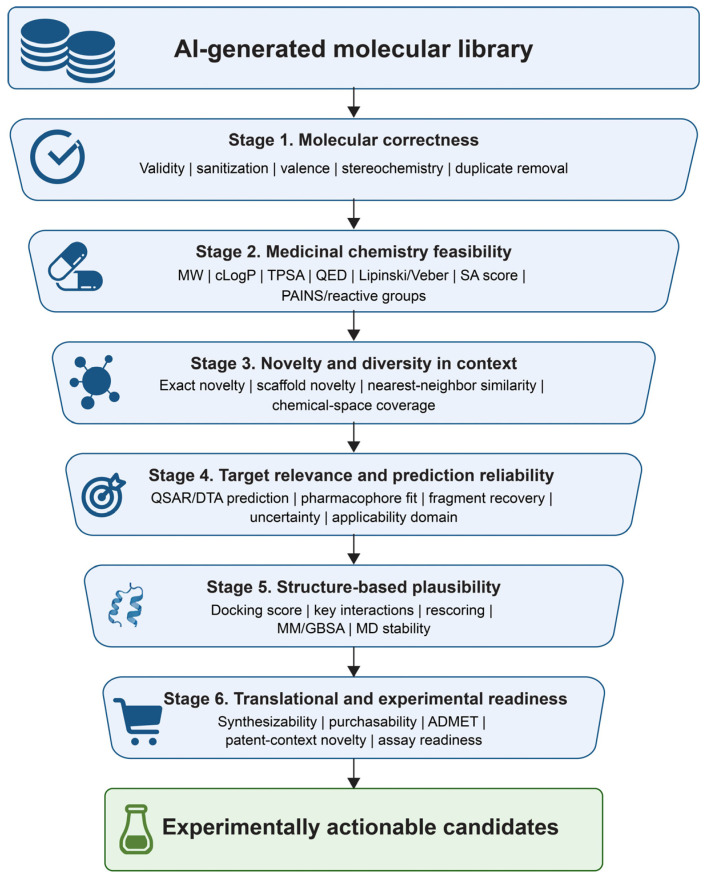
Conceptual overview of the staged evaluation framework for AI-generated molecules in drug discovery, progressing from molecular correctness to translational readiness across six evidence layers.

The evaluation of AI-generated molecules should therefore not depend on a single metric or a small set of generic generative statistics. The individual metrics and analyses discussed in this review are not proposed as new measures. Many of them are already established in molecular generation benchmarks, medicinal chemistry filtering, QSAR validation, docking evaluation, pose validation, molecular simulation, ADMET prediction, and synthesis planning. The contribution of this review is to bring these dispersed practices together into a staged, failure-aware, and evidence-graded framework. The framework begins with molecular correctness and then considers medicinal chemistry feasibility, contextual novelty and diversity, target relevance and prediction reliability, structure-based plausibility, and translational readiness. It links each layer of evaluation to the strength of claims that can reasonably be made for AI-generated molecules. Figure 1 summarizes this staged evaluation concept. Rather than providing another taxonomy of molecular generative architectures, this review focuses on how generated molecules should be judged after they have been produced. We discuss common evaluation metrics, their interpretive limits, frequent failure modes, and practical reporting considerations. The central argument is that the evaluation of AI-generated molecules should move beyond validity and novelty and toward multilayered evidence of practical usefulness.

## 2. AI-Driven Molecular Generation: Models and Evaluation in Current Practice

Evaluation criteria for AI-generated molecules depend strongly on the molecular representation, generation strategy, and optimization objective. Sequence-based, graph-based, and pocket-conditioned three-dimensional models produce different forms of molecular output, so the same metric may have different implications across model classes. For example, a docking score is more informative for a pocket-conditioned three-dimensional generator than for an unconditional sequence-based model, whereas syntactic validity is largely guaranteed under some recent string representations. This section outlines the main model families, recent translational milestones, and common reporting practices in widely cited molecular generation studies. Several comprehensive technical reviews complement the present discussion [16,17,18,19].

### 2.1. Molecular Representations and Generative Architectures

Molecular representations define what a generator outputs and therefore determine which validity and similarity checks are appropriate. Three families dominate current practice. One-dimensional string representations encode molecular graphs as linear character sequences. Simplified molecular-input line-entry system (SMILES) has long been the default representation [20], but minor syntactic errors can produce invalid molecules, which has motivated alternatives such as DeepSMILES and, most prominently, self-referencing embedded strings (SELFIES) [21]. In SELFIES, every syntactically valid string maps to a chemically valid molecule. Models based on SELFIES can therefore achieve almost perfect syntactic validity under appropriate token constraints, shifting the evaluation question from validity itself to whether the generated chemistry is useful. Two-dimensional graph representations describe molecules as labeled graphs of atoms and bonds and are typically generated atom by atom, fragment by fragment, or in a single step through graph neural networks [22,23,24,25]. Three-dimensional representations specify atomic coordinates and are central to structure-based de novo design. Recent 3D generators condition generation directly on a protein pocket and output ligand structures in three-dimensional space [26,27,28,29], where validity must extend beyond graph-level sanitization to include geometric and physical plausibility, a concern made explicit by PoseBusters [30]. Earlier hybrid systems such as MolAICal also attempted to combine artificial intelligence with classical structure-based design procedures for protein-target-oriented 3D drug design, using pocket information, ligand generation, docking, and medicinal chemistry filters to support candidate prioritization [31].

Generative architectures for molecules can be grouped into several broad families. Recurrent and transformer-based language models learn next-token distributions over string representations and remain among the most widely used architectures in practical pipelines, partly because they integrate naturally with reinforcement learning (RL). REINVENT is a representative example that fine-tunes a pretrained sequence model toward user-defined objectives [3], and related language-model-based approaches have been extended to scaffold decoration, fragment growing, and pocket-conditioned generation [29,32]. Variational autoencoders (VAE) learn a continuous latent space from which new molecules can be sampled by interpolation or property-guided traversal. JT-VAE [22] and GENTRL [33] are influential examples. Generative adversarial networks, exemplified by MolGAN [23], formulate molecular generation as an adversarial competition between a generator and a discriminator. Normalizing-flow-based molecular graph generators, containing GraphAF [25] and MoFlow [34], utilize invertible transformations to model molecular graph distributions. This design enables tractable likelihood-based training and efficient molecular generation. Three-dimensional generators also cover several subtypes. Autoregressive 3D approaches, such as Pocket2Mol [26], place atoms sequentially inside a protein pocket, whereas diffusion-based models have recently become a major family for 3D molecular generation. Part of the reason is that they can model continuous atomic coordinates while respecting Euclidean symmetries through networks equivariant to the special Euclidean group in three dimensions (SE(3)). Equivariant diffusion models (EDM) [35], TargetDiff [27], DiffSBDD [28], and DiffMC-Gen [36] illustrate this direction. DiffMC-Gen further extends diffusion-based molecular generation by integrating discrete and continuous molecular features and by using multi-objective constraints to jointly consider binding affinity, drug-likeness, synthesizability, and toxicity. Hybrid approaches that combine geometric deep learning with language modeling, such as Lingo3DMol [29], reflect a recent move toward integrating representational strengths across model families.

From an evaluation standpoint, the relevant point is that different architectures generate different objects, including strings, graphs, and 3D structures. They also provide different validity guarantees and allow different optimization mechanisms, all of which shape which evaluation metrics are meaningful. The main families and their evaluation-relevant features are summarized in Table 1.

### 2.2. Goal-Directed Generation and Translational Milestones

Beyond unconditional distribution learning, most practical workflows aim to bias generation toward molecules that meet specific design objectives. Reinforcement learning is one of the most common strategies. REINVENT pioneered the use of reinforcement learning to fine-tune a pretrained sequence-based generator toward user-defined property or activity rewards [3], and the broader ReLeaSE framework demonstrated reinforcement-learning-guided design using biological activity rewards [37]. Target-focused reinforcement-learning strategies have also been extended to kinase inhibitor design. For example, KinGen integrates reinforcement learning, transfer learning, and an affinity-prediction-based reward module to guide the generation of kinase-oriented molecules with predicted target relevance [38]. Conditional generation provides another route, in which target properties, scaffolds, fragments, or protein pockets are provided to the generator as conditioning inputs [39]. Structure-based conditioning represents a further class of strategies, in which 3D generators are trained or sampled with an explicit target pocket and produce molecules placed directly within the binding site [26,27,28,29]. Docking- and pharmacophore-guided generation represents another form of target-directed design. COMG uses docking scores and three-dimensional pharmacophore matching within a conditional variational autoencoder framework, with a scaffold memory unit introduced to help maintain structural diversity during optimization [40]. Across these goal-directed strategies, a recurring concern, and one that is central to the evaluation discussion in later sections, is that the reward or conditioning signal is only a proxy for the scientific objective of interest. A QSAR score is a proxy for biological activity, a docking score is a proxy for binding affinity, QED is a proxy for drug-likeness, and the SA score is a proxy for synthetic accessibility. Strong optimization pressure can move these proxies into regions where they no longer reflect the intended goal, a problem often described as reward exploitation or reward hacking and discussed in detail in later sections.

Practical impact has advanced alongside methodological progress. The 2019 GENTRL study used a VAE-based generator with tensor-decomposition priors and reinforcement-learning fine-tuning to design discoidin domain receptor 1 (DDR1) kinase inhibitors, two of which were validated in cell-based assays, while one showed favorable pharmacokinetics in mice [33]. More recently, ISM001-055, now named rentosertib, a small-molecule TRAF2- and NCK-interacting kinase (TNIK) inhibitor with anti-fibrotic activity, has progressed from AI-enabled discovery to a published Phase 2a clinical evaluation, providing a prominent example of AI-enabled molecular design reaching prospective clinical testing [41,42]. Projects in other industries have already reported some preclinical or early clinical candidate drugs derived from generative pipelines, although the extent of methodological details published in these reports varies greatly. These milestones show that AI-driven molecular generation can contribute to real discovery workflows. At the same time, they reveal a gap between model development and experimental follow-up. The number of generative models released each year far exceeds the number of experimentally validated generated compounds. The depth of validation also varies greatly between studies. These examples therefore underscore that translational claims require evidence beyond benchmark-level generative performance.

### 2.3. How Generated Molecules Are Evaluated in Current Practice

Evaluation practice has not kept pace with the diversification of model architectures. Across widely cited methodological papers covering sequence-based, graph-based, and three-dimensional pocket-conditioned models, including GuacaMol [1], MOSES [2], REINVENT [3], CharRNN [43], JT-VAE [22], GCPN [24], GENTRL [33], Pocket2Mol [26], TargetDiff [27], and DiffSBDD [28], the reported evidence remains concentrated in a small number of metric families. Conventions established by early benchmark papers continue to determine what much of the subsequent literature reports.

Validity, uniqueness, novelty against the training set, and pairwise diversity remain the most consistently reported metrics for sequence- and graph-based generators, largely following the conventions established by GuacaMol and MOSES. MOSES additionally introduced internal diversity (IntDiv_1_ and IntDiv_2_), nearest-neighbor similarity, and fragment- and scaffold-level distributional comparisons, which are useful for detecting mode collapse and memorization [2]. These distributional metrics are reported mainly when studies benchmark explicitly against MOSES. In goal-directed and structure-based studies, they are much less frequently revisited. The Fréchet ChemNet Distance (FCD) [44], introduced as a general distributional check, shows a similar pattern of use.

Scalar drug-likeness measures, including QED, the synthetic accessibility score, Lipinski’s rules, and PAINS filters, appear in nearly all studies that evaluate the chemistry of generated molecules, often as the only chemistry-aware metrics reported [6,7,8,10]. JT-VAE and GCPN report penalized logP or QED as primary objectives, while subsequent pocket-conditioned generators such as Pocket2Mol, TargetDiff, and DiffSBDD continue to report QED and SA alongside docking scores. These scalar measures are convenient and easy to compare, but their thresholds, appropriate contexts, and known limitations are rarely discussed. For example, Lipinski’s Rule of Five provides useful first-pass guidance for oral drug-likeness, but it should not be applied as a rigid exclusion rule for all generated molecules [6].

For three-dimensional pocket-conditioned generators, AutoDock Vina [45] or QuickVina [46] docking scores have largely replaced distribution-aware metrics as the headline result. Pocket2Mol, TargetDiff, and DiffSBDD all report Vina scores against CrossDocked2020 [47] references, and most also report the fraction of generated molecules predicted to bind at least as well as the crystallographic reference ligand [26,27,28]. Comparatively few studies in this class report graph-level validity, novelty against the training set, or distributional comparisons with the test set. Recent independent analyses have shown that Vina scores correlate strongly with molecular size and can be inflated by simply adding atoms, while more stringent measures such as specific binding ability have not improved at the same rate [48]. PoseBusters-style geometric validity checks [30] are beginning to appear, but they are not yet standard practice. A recent TarPass benchmark further reinforces this concern by systematically evaluating target-aware de novo molecular generation across non-3D, 3D in situ, and optimization-based paradigms. Rather than relying only on docking scores, TarPass assesses whether generated molecules recover meaningful protein–ligand interactions, remain chemically plausible and drug-like, and outperform realistic random baselines, thereby highlighting the gap between favorable structure-based scores and genuine target-aware molecular design [49]. Accordingly, raw docking scores should be interpreted with size-aware controls, reference or random baselines, and pose-level inspection rather than used as standalone rankings [48,49].

Experimental follow-up remains rare. The GENTRL DDR1 program [33] and the ISM001-055 clinical disclosure [41,42] are widely cited precisely because such reports are uncommon, and both come from a single industrial group. Most methodological studies stop at computational evaluation, leaving the published evidence base for molecular generative models dominated by in silico claims whose translation has not been independently tested. This imbalance is one of the central motivations for the staged, evidence-graded evaluation framework developed in the rest of this review. A concise summary of common evaluation metrics and their interpretation limits is provided in Table S1, while the following sections discuss these evidence layers in greater detail.

## 3. Basic Generative Metrics: Necessary but Insufficient

Validity, uniqueness, novelty, and diversity are the metrics most consistently reported across molecular generation studies. They characterize basic properties of generated molecules and provide a first assessment of model behavior. However, these metrics alone do not establish chemical feasibility, biological relevance, or practical utility.

### 3.1. Validity and Chemical Correctness

In SMILES-based generation, validity is most often defined as the fraction of generated strings that can be parsed into molecular graphs by cheminformatics toolkits, reflecting the role of SMILES as a machine-readable line notation [20]. For example, “CCO” is a valid SMILES string for ethanol, whereas malformed strings such as “C1CC” with an unclosed ring, or strings with unmatched brackets or invalid valence patterns, may fail parsing or sanitization. In graph-based generation, validity also requires consistency in valence, atom-type constraints, and stereochemical handling. For three-dimensional generators, validity must extend further to geometric and physical plausibility, including bond lengths, bond angles, planarity, and steric feasibility. Graph-level sanitization is no longer sufficient in this setting. PoseBusters-type checks are particularly relevant where the distinction between topological and geometric correctness becomes apparent [30].

Validity should be understood as a minimum entry requirement instead of evidence of practical value. A parsable and sanitized structure may still correspond to a molecule with undesirable substructures, difficult stereochemistry, poor physicochemical properties, or features that make synthesis or testing unsuitable. Validity also depends on preprocessing and standardization choices. Salt removal, charge neutralization, tautomer standardization, stereochemical normalization, or post hoc correction can all affect the reported validity rate. Studies should therefore report the molecular representation, parsing toolkit and version, standardization rules, canonicalization procedure, and whether invalid molecules were corrected or discarded.

### 3.2. Uniqueness and Sampling Redundancy

Uniqueness measures whether a generator repeatedly produces the same molecules. It is usually calculated after canonicalization, so that different SMILES strings representing the same molecular graph are treated as duplicates. A model that outputs a large number of structures but contains only a small number of unique molecules has limited effective exploration. Uniqueness is also strongly dependent on sample size. MOSES reports this metric at multiple sample sizes, such as Uniqueness@1000 and Uniqueness@10000, because uniqueness tends to decline as the number of generated molecules increases [2]. A model that appears non-redundant at small sample sizes may still collapse into a narrow output distribution under larger sampling.

Uniqueness is nevertheless a narrow metric. High uniqueness means that exact duplicates are uncommon, but it does not guarantee chemical diversity, scaffold innovation, or useful exploration. A generator can produce many unique molecules through small substituent changes around the same core scaffold. In such cases, the output may appear non-redundant at the exact-structure level while remaining narrow at the scaffold level. Low uniqueness is not always a failure either. In lead optimization, repeated generation of close analogues around a hit may be desirable. Uniqueness should therefore be interpreted in relation to the design goal and sample size.

### 3.3. Novelty and the Ambiguity of “Newness”

Novelty is commonly defined as the fraction of generated molecules absent from a defined reference set. The choice of reference set determines the question being asked. Novelty against the training set asks whether the model memorizes its training data, whereas novelty against an external library such as ChEMBL [50], ZINC [51], or PubChem [52] asks whether the generated molecules are new relative to known chemistry more broadly. These two definitions answer different questions and should not be conflated.

Exact structural absence captures only one form of newness. A molecule may be absent from the training set while still closely resembling a known active compound, a patented structure, or a common scaffold with minor substitutions. Such a molecule would pass an exact-match novelty test, yet it may contribute little medicinal chemistry novelty. Conversely, a molecule structurally close to a known active compound can still be valuable if it improves potency, selectivity, solubility, metabolic stability, or synthetic accessibility. Novelty should therefore be interpreted according to the design objective rather than treated as inherently beneficial.

A more informative assessment distinguishes several levels of novelty. Exact novelty asks whether the generated molecule is absent from a defined reference set. Scaffold novelty asks whether the core framework differs from known frameworks, often using Bemis–Murcko scaffold definitions [9]. Analogue-level novelty asks whether the molecule is close to known actives, patented compounds, or training molecules in fingerprint or descriptor space. Memorization checks can add further information as a generator may avoid exact duplication while still reproducing training fragments or scaffolds in nearly identical proportions. MOSES fragment- and scaffold-level distributional comparisons can help detect this form of fragment-level memorization [2].

Novelty also needs to be considered together with target relevance and feasibility. If novelty is optimized without constraints, the model may generate unusual molecules that are far from known chemical space but difficult to synthesize or unlikely to bind the intended target. Conservative molecules may be less novel, but they can be more suitable for rapid experimental follow-up. This trade-off explains why novelty alone is not a reliable success criterion in drug discovery.

### 3.4. Diversity and Scaffold-Level Exploration

Diversity describes how broadly generated molecules cover chemical space. Extended-connectivity fingerprints with Tanimoto similarity remain commonly used for pairwise diversity assessment [53,54], complemented by scaffold counts and scaffold-frequency distributions. Distribution-aware metrics provide a more nuanced view than scalar diversity averages. MOSES metrics such as internal diversity, nearest-neighbor similarity, and fragment- and scaffold-level distributional comparisons help distinguish within-set diversity from distributional shift relative to a reference set [2].

Scaffold-level analysis is particularly important because medicinal chemistry often reasons in terms of core frameworks rather than complete structures alone. The Bemis–Murcko framework separates molecules into ring systems, linkers, side chains, and molecular frameworks, and remains a common basis for scaffold analysis in drug-like chemical space [9]. In molecular generation, scaffold diversity can show whether a model explores different core structures or mainly decorates a small number of scaffolds with different substituents.

High Tanimoto-based diversity is not automatically desirable. A generator can achieve high diversity by producing molecules that are chemically scattered, difficult to synthesize, outside useful property ranges, or irrelevant to the biological objective. This problem is especially pronounced in target-directed design. A highly diverse generated set with poor predicted activity, poor property balance, or implausible binding modes may be less useful than a moderately diverse set enriched with target-relevant and synthetically tractable candidates. Diversity should therefore be reported not only for the raw generated set, but also after key filtering steps such as validity filtering, medicinal chemistry filtering, target-aware scoring, or structure-based prioritization.

Distributional similarity scores such as the Fréchet ChemNet Distance compare latent-feature distributions between generated and reference molecules using a pretrained ChemNet representation [44]. FCD is useful as a general distributional check that goes beyond simple scaffold counts. However, it remains sensitive to the representation on which it depends, and apparent improvements in FCD do not always correspond to chemically meaningful changes in the generated distribution. FCD is therefore most informative when reported alongside chemically interpretable metrics such as scaffold counts, property distributions, and nearest-neighbor similarity.

### 3.5. Interpretation Limits of Basic Generative Metrics

Reproducibility requires studies to specify, for each metric, the reference set used, the fingerprint and similarity measure applied, the sample size at which the metric was computed, and whether the metric was computed before or after filtering. Without these details, basic generative metrics are easy to compare formally but difficult to compare meaningfully across studies. As Handa et al. recently observed, retrospective validation alone cannot fully establish the practical value of newly generated molecules, whereas prospective validation remains expensive and subject to selection bias [55]. The remaining sections of this review address this gap one layer at a time.

## 4. Medicinal Chemistry Feasibility Beyond Generative Statistics

Generative metrics indicate whether a model can produce valid, unique, novel, and diverse molecules, but they do not show whether those molecules are credible starting points for drug discovery. A generated structure may pass basic cheminformatics checks and still have poor physicochemical properties, difficult synthetic routes, reactive groups, assay-interfering motifs, or little room for later optimization. Medicinal chemistry feasibility should therefore be assessed once basic generative quality has been established. This layer of evaluation does not prove that a molecule will become a drug. It asks whether the generated structure is suitable for further computational prioritization, synthesis planning, or experimental testing.

### 4.1. Drug-likeness and Physicochemical Property Balance

Drug-likeness is commonly used to describe whether a compound has physicochemical properties similar to those of known drugs or successful drug discovery candidates. In molecular generation studies, this profile is usually assessed using molecular weight (MW), lipophilicity, hydrogen-bond donors (HBDs) and acceptors (HBAs), topological polar surface area (TPSA), rotatable bonds, QED, and rule-based filters. These descriptors are useful because many early discovery failures arise not only from insufficient target potency, but also from poor solubility, permeability, metabolic stability, and other developability-related properties.

The Lipinski Rule of Five remains one of the most widely used empirical guidelines for oral drug-likeness. Lipinski et al. reported that poor absorption or permeation is more likely when a compound has more than 5 hydrogen-bond donors, more than 10 hydrogen-bond acceptors, a molecular weight above 500, and a calculated logP above 5 [6]. The rule was derived from orally active compounds and should be read as a risk indicator, not as an absolute exclusion criterion. For AI-generated small molecules intended for conventional oral drug discovery, compliance with the Rule of Five is still a useful first-pass check, but compounds outside the classical range should not be rejected mechanically.

Other property guidelines provide complementary information. Veber et al. found that lower molecular flexibility, measured by the number of rotatable bonds, together with lower polar surface area or total hydrogen-bond count, was associated with better oral bioavailability in their dataset [56]. This observation is relevant to molecular generation because some models produce molecules with acceptable validity and novelty but excessive flexibility, polarity, or molecular size. Such compounds may appear chemically diverse while having a lower chance of favorable absorption or permeability.

QED is also widely used in molecular generation. Bickerton et al. proposed QED as a quantitative estimate of drug-likeness by combining several molecular property desirability functions into a single score [7]. Its main advantage is that it is easy to compute and convenient for comparing generated sets. QED, however, summarizes similarity to the property profile of known drugs and does not assess target engagement, selectivity, safety, synthetic route, or clinical utility. QED is therefore most informative when reported together with individual physicochemical descriptors rather than used as the sole medicinal chemistry metric.

Average property values can also conceal problematic subsets. A generated set may show an acceptable average QED or average molecular weight, but it still contains many molecules with extreme lipophilicity, excessive polarity, or poor size control. Leeson and Springthorpe discussed how drug-like concepts have influenced decision-making in medicinal chemistry. They also pointed out that compounds synthesized in discovery programs may deviate from the range of properties of successful oral drugs and clinical compounds [57]. For AI-generated molecules, property distributions, percentile ranges, and comparisons with known active compounds or relevant screening libraries are more informative than average values alone.

### 4.2. Synthetic Accessibility and Chemical Tractability

Synthetic accessibility (SA) is a central issue for AI-generated molecules because a computationally attractive structure has limited value if it cannot be synthesized or obtained for testing. Many molecular generation studies estimate synthetic feasibility using the synthetic accessibility score proposed by Ertl and Schuffenhauer [8]. This score combines fragment contributions from known molecules with a molecular complexity penalty and provides a rapid estimate of how difficult a molecule may be to synthesize. It is useful for large-scale filtering because it can be applied to many generated structures at low computational cost.

The SA score is still only an approximate descriptor. It does not provide a synthetic route, reagent availability, reaction yield, purification difficulty, cost, scalability, or information about stereochemical control. A molecule with a favorable SA score may still be difficult to synthesize in practice, whereas a molecule with a less favorable score may be accessible through a known route or a specialized synthetic strategy. The SA score should therefore be used as an early warning signal rather than as proof of synthesizability.

Generated molecules selected for experimental follow-up usually require additional checks. These may include retrosynthetic analysis, purchasability search, building-block availability, route length, stereochemical complexity, and expert review by medicinal chemists. Retrosynthetic planning has advanced substantially through data-driven and neural-symbolic methods. Segler et al., for example, combined neural networks with symbolic artificial intelligence for chemical synthesis planning, illustrating how computational tools can support route proposal and prioritization [58]. Because practical feasibility depends on reaction conditions, selectivity, purification, reagent costs, and laboratory constraints, proposed routes still require chemical review.

A related problem is that generative models may produce structures that are formally valid but chemically unattractive. Examples include unusual ring systems, unstable linkages, highly strained motifs, excessive stereochemical complexity, and functional groups that complicate synthesis. These features may not be captured by validity, novelty, QED, or a single SA score. Synthetic tractability should therefore be evaluated together with structural inspection, property filters, retrosynthetic evidence, and chemical-alert analysis, especially when generated molecules are presented as testable candidates.

### 4.3. Structural Alerts, Reactive Groups, and Undesirable Chemotypes

Generated molecules should also be screened for problematic substructures. Some functional groups may be reactive, unstable, toxic, redox-active, metal-chelating, or prone to assay interference. Others may behave as frequent hitters in biochemical or cellular assays and produce false-positive activity signals. In screening-library design, compound selection often includes filters for unwanted groups and undesirable motifs. Brenk et al. described unwanted-group rules and lead-like selection criteria for assembling screening libraries in neglected-disease drug discovery, showing that chemical tractability and assay suitability should be considered before biological testing [59].

PAINS filters are commonly used to identify substructures associated with pan-assay interference. Baell and Holloway introduced PAINS substructure filters to remove compounds that appeared as frequent hitters in multiple high-throughput screening assays [10]. This problem is relevant to molecular generation because a model optimized only for predicted activity, docking score, or another surrogate objective may generate molecules with problematic motifs unless these features are explicitly penalized or removed.

Structural alerts should still be applied with care. A PAINS or reactive-group flag does not by itself prove that a compound is inactive, toxic, or unusable. Its meaning depends on the chemical context, assay format, concentration, mechanism of action, and availability of orthogonal evidence. Baell and Walters later emphasized that assay-interfering compounds can waste substantial resources in drug discovery, but the appropriate response is careful triage rather than blind filtering [60]. Recent discussions of nuisance compounds in cellular assays also show that assay interference is context-dependent and may require counterscreens or orthogonal assays [61].

For AI-generated molecules, structural-alert reporting helps readers judge whether the final generated set is chemically reasonable. Useful reporting may include the number and proportion of generated molecules marked by PAINS filters, reactive group filters, toxicophore alerts, or internal medicinal chemistry rules. If flagged molecules are retained, the rationale should be indicated. If they are removed, the filtering rules should be described clearly.

### 4.4. Lead-likeness Versus Drug-likeness in Early Discovery

A common limitation when evaluating AI-generated molecules is the tendency to regard drug-likeness as the only desirable property profile. In early discovery, the aim is often to generate hit-like or lead-like starting points for optimization rather than final drug-like molecules. Notably, a compound that is already large, lipophilic, and highly functionalized may have little room for further optimization. By contrast, a smaller and less complex molecule with moderate potency may be more valuable if it supports systematic exploration of structure–activity relationships.

Hann and Oprea discussed lead-likeness and suggested that early lead compounds should have lower molecular complexity and physicochemical properties suitable for optimization, rather than merely resembling marketed drugs [62]. This distinction is especially relevant to AI-generated molecules. When a model is optimized too strongly for drug-likeness scores, it may produce compounds that are statistically similar to drugs but poorly suited as medicinal chemistry starting points. If a model is optimized only for predicted potency, it may generate large, lipophilic, or poorly soluble compounds with limited developability.

Ligand efficiency is another useful concept in early discovery because it relates binding potency to molecular size. Hopkins et al. introduced ligand efficiency to assess whether potency is gained efficiently, instead of mainly through increased molecular weight [63]. For AI-generated molecules, ligand efficiency can help determine whether predicted activity is accompanied by an acceptable size and property profile. This measure is particularly useful when comparing small, simple candidates with larger molecules that receive high activity scores but leave less room for optimization.

Three-dimensionality and saturation can also support medicinal chemistry evaluation. Lovering et al. reported that higher saturation, measured by the fraction of sp3 carbons, and the presence of chiral centers were associated with progression from discovery through clinical testing to drugs in their analysis [64]. This does not mean that high fraction of sp3-hybridized carbon atoms (Fsp3) is always preferable, but it suggests that flat and highly aromatic compounds may carry risks related to solubility, selectivity, and promiscuity. For generated molecules, Fsp3, aromatic ring count, and stereochemical complexity can therefore serve as supportive descriptors when assessing whether a generator produces chemically tractable and optimizable structures.

For early hit discovery, useful reporting should include both drug-likeness and lead-likeness indicators. These may include molecular weight, calculated logarithm of the octanol/water partition coefficient (cLogP), ligand efficiency, topological polar surface area, rotatable bonds, aromatic ring count, Fsp3, stereocenter count, structural alerts, and synthetic accessibility. No single metric is sufficient, but these descriptors together can show whether generated molecules have a reasonable balance among potency-oriented features, chemical tractability, and optimization potential.

## 5. Target Relevance and Biological Plausibility

After basic generative quality and medicinal chemistry feasibility have been assessed, the next question is whether the generated molecules are relevant to a biological target or disease context. This step is critical because a molecule can be valid, novel, drug-like, and synthetically accessible while still having little connection to the intended pharmacological objective. In practical drug discovery, generated molecules are rarely useful simply because they occupy an acceptable region of chemical property space. They also need evidence of target engagement, pathway relevance, phenotypic effect, or mechanistic plausibility.

Target relevance is usually assessed using surrogate computational evidence. Common approaches include ligand-based activity prediction, drug–target interaction or affinity prediction, similarity analysis against known active compounds, pharmacophore matching, fragment recovery, docking-based evaluation, and multi-objective scoring. These methods are useful because they allow large generated libraries to be prioritized before experimental testing. Their outputs, however, should be interpreted according to the reliability and independence of the evidence used. A molecule predicted to be active is not the same as an experimentally active molecule, and a high target-aware score does not guarantee potency, selectivity, cellular activity, or developability.

### 5.1. Similarity, Pharmacophore, and Fragment-Based Analyses

Similarity-based evaluation offers a simple way to assess whether generated molecules are related to known active chemical space. Molecular similarity is often calculated using fingerprints and Tanimoto similarity. This analysis can indicate whether a generated set contains close analogues of known actives, possible scaffold-hopping candidates, or chemically distant structures. Because similarity analysis is easy to compute and interpret, it is useful as an early target-relevance check.

Similarity, however, is not equivalent to biological activity. Compounds that are highly similar to known actives may be more likely to share activity, but this relationship is not guaranteed [65]. Small structural changes can strongly affect potency, selectivity, metabolic stability, or binding mode, giving rise to activity cliffs, where structurally similar compounds show unexpectedly large differences in biological activity [66]. Conversely, compounds with low two-dimensional similarity may still bind to the same target if they preserve key interaction features. Similarity should therefore not be used as a standalone surrogate for activity.

Pharmacophore analysis provides a more functional view than simple structural similarity. A pharmacophore describes spatial arrangements of features such as hydrogen-bond donors, hydrogen-bond acceptors, aromatic rings, hydrophobic centers, or charged groups that are considered important for molecular recognition. In generative design, pharmacophore matching can help assess whether generated molecules preserve interaction hypotheses derived from known ligands or protein–ligand complexes. Tools such as LigandScout have long been used to derive three-dimensional pharmacophores from protein-bound ligands and to support virtual screening at the level of interaction features rather than exact chemical identity [67].

The value of pharmacophore matching depends on the quality and specificity of the underlying hypothesis. A restrictive pharmacophore may favor trivial analogues and reduce scaffold diversity, whereas a loose pharmacophore may accept molecules that do not form the intended interactions. Fragment-based or motif-recovery analysis can provide complementary information. This analysis shows whether generated molecules preserve substructures or scaffold elements associated with known active compounds. High fragment recovery may indicate useful target-related learning, but it may also reflect conservative reproduction of known chemistry. Low fragment recovery may indicate structural innovation, but it may also signal a loss of target relevance. These analyses are most informative when interpreted together with novelty, similarity, predicted activity, and medicinal chemistry feasibility.

### 5.2. Activity Prediction and Target-Aware Scoring

One common way to assess target relevance is to use a predictive model that estimates biological activity, binding affinity, or interaction probability. In ligand-based settings, these models are usually QSAR classifiers or regressors trained on known active and inactive compounds. In target-aware settings, DTI or DTA models may encode both the molecule and the protein target, allowing the same scoring model to be applied across multiple targets. DeepDTA is a representative early example of a sequence-based deep learning model for drug–target binding affinity prediction, using SMILES strings for compounds and amino acid sequences for proteins as inputs [4]. Related models have further extended this idea by using graph neural networks to represent molecular structures, as in GraphDTA [68], or transformer-based architectures to model drug–target interactions, as in MolTrans [69].

The appeal of target-aware scoring is clear. It is much faster than experimental screening and is usually less expensive computationally than docking or molecular simulation. It can also be incorporated into generative workflows as a reward function, filter, or ranking model. In reinforcement learning-based molecular generation, for example, a predictive score can guide the model toward molecules that satisfy a desired objective. REINVENT is an influential example of this strategy, showing that a pretrained sequence-based molecular generator can be fine-tuned toward user-defined molecular properties or predicted biological activity through reinforcement learning [3].

Activity prediction should still be treated as a prioritization tool, not as direct evidence of real activity. Predictive models are constrained by the quality, coverage, and bias of their training data. They may become unreliable when applied to generated molecules that differ from the training distribution. This issue is particularly relevant in de novo design, where the goal is often to explore new regions of chemical space. For this reason, studies that utilize activity predictors should report the endpoint definition, training data, validation strategy, activity threshold, and relationship between predicted scores and molecular properties.

The validation setting should also match the intended use of the predictor. Random splits may overestimate performance when close analogues appear in both training and test sets. Scaffold-based, temporal, or external validation can provide a more informative estimate of prospective performance, especially when the model is expected to score newly generated chemistry [70,71]. Inactive compounds and decoys should also be defined carefully. In this context, decoys are presumed inactive or non-binding molecules used as negative controls, ideally selected to avoid trivial differences from active compounds in simple physicochemical properties. Poorly matched decoys may make a model appear more reliable than it is [72]. When a target-aware model is used to score generated molecules, useful comparisons involve known active ligands, appropriate inactive or decoy sets, molecules generated by an unoptimized prior model, and molecules outside the optimization reward. These comparisons help determine whether high target-aware scores reflect meaningful target relevance or artifacts of the scoring function.

### 5.3. Applicability Domain, Uncertainty, and Predictor Bias

A major risk in target-aware evaluation is that the scoring model may be applied outside its reliable domain. This problem is well recognized in QSAR modeling. QSAR validation studies emphasize that predictive models should be used only within a defined applicability domain, namely the chemical or descriptor space adequately represented by the training data, where predictions can be considered reliable [73,74]. Tropsha also emphasized that QSAR models require careful validation, including external validation and applicability-domain analysis, before they are used for prediction [5].

This issue is especially important for AI-generated molecules. Generative models are often designed to move beyond known chemistry, whereas predictive models are usually trained on known chemistry. This creates a tension. The more novel a generated molecule is, the less certain its activity prediction may be. Evaluation should therefore ask not only whether a molecule receives a high predicted score, but also whether it lies within the model’s reliable prediction domain.

Uncertainty estimation can help, although it is not a complete solution. Possible approaches include ensemble variance, distance to the training set, conformal prediction, Bayesian methods, and calibration analysis. In practice, even a simple comparison between generated molecules and training compounds in descriptor or fingerprint space can be informative. If highly scored generated molecules are far outside the training domain, their predictions should be treated as hypotheses requiring further validation rather than as strong evidence of activity.

Predictor bias is another concern. Activity datasets are often unevenly distributed across targets, chemical series, assay formats, and potency ranges. A model may learn dataset-specific patterns rather than generalizable target-binding principles. It may favor chemical motifs that are overrepresented among known actives, or fail to recognize novel scaffolds that lack close training examples. In multi-target models, targets with more data may also be modeled more reliably than underrepresented targets. When target-aware scores are used to evaluate generated molecules, the training data, validation strategy, applicability domain, and uncertainty information should therefore be described clearly.

### 5.4. Reward Exploitation in Target-Directed Generation

When target-aware scoring is used only for post hoc ranking, its limitations are serious but relatively contained. The risk becomes larger when the same score is used as an optimization objective during generation. In reinforcement learning or other goal-directed optimization settings, the model may learn to exploit weaknesses in the scoring function. This problem is often described as reward exploitation, reward hacking, or predictor hacking in goal-directed molecular generation [75,76,77,78]. Generated molecules may achieve high computational scores without becoming more meaningful drug discovery candidates.

This issue is particularly relevant because molecular design objectives are usually proxies. A QSAR score is a proxy for biological activity. A docking score is a proxy for binding favorability. QED is a proxy for drug-likeness. SA score is a proxy for synthetic accessibility. Strong optimization pressure can push these proxies into regions where they no longer reflect the intended objective. Benchmarking studies such as GuacaMol are valuable because they provide standardized goal-directed tasks, but benchmark performance still needs to be distinguished from real discovery success.

Several safeguards can reduce the risk of reward exploitation. The reward should include basic chemical constraints, such as validity, property ranges, and filters for undesirable substructures. Optimization should also preserve some relationship to a learned chemical prior, rather than allowing unconstrained movement toward high-scoring but unrealistic structures. Generated molecules should then be evaluated using orthogonal methods that were not part of the reward function. Molecules optimized by a DTA predictor, for example, can be examined by docking, pharmacophore analysis, applicability-domain assessment, or experimental assays where available. Molecules optimized by docking can be checked using medicinal chemistry filters, pose inspection, rescoring, MD-based analyses, or independent activity prediction. Reporting should include not only the best-scoring molecules but also score distributions, reward components, and failure rates after filtering.

Multi-objective reward design can help, but it should be used transparently. Combining predicted activity, QED, SA score, novelty, and diversity may produce more balanced molecules than optimizing a single score. The resulting function can still be arbitrary if the weights are not justified. A molecule with a high composite score may fail for a simple reason, such as poor solubility, reactive groups, or lack of a synthetic route. Multi-objective scoring should therefore be accompanied by clear reporting of each component, rather than only the final aggregate score.

## 6. Structure-Based Assessment of AI-Generated Molecules

For target-directed molecular generation, chemical feasibility and target-aware scores are not sufficient unless they can be linked to a plausible mode of molecular recognition. A generated compound may have acceptable physicochemical properties and a high predicted activity score, yet still fail to fit the intended pocket, form unrealistic contacts, or adopt a strained binding pose. Structure-based assessment adds an important layer of evidence for AI-generated molecules, especially when an experimental protein structure, a homology model, or a high-confidence predicted structure is available. Recent reviews of generative molecular design and AI-assisted protein-structure prediction have highlighted both the rapid expansion of computational design pipelines and the need to avoid overinterpreting computational outputs [18,19,79]. Structure-based plausibility should therefore be viewed as prioritization evidence rather than direct proof of binding, potency, or translational readiness.

Structure-based methods should not be viewed as replacements for biochemical or biophysical assays. Their main role is to examine whether generated molecules are compatible with the known or proposed structural constraints of a target. In practice, these methods address several related but distinct questions. Docking examines pocket compatibility and provides an initial ranking. Pose inspection evaluates whether the predicted binding mode is chemically interpretable. Rescoring and MM/GBSA can support relative prioritization within a filtered set. Molecular dynamics can test short-timescale pose stability and, when combined with appropriate free-energy methods, can contribute to affinity-oriented assessment for a limited number of prioritized compounds. These layers provide stronger evidence than docking scores alone, but their interpretation still depends on protocol quality, sampling, and appropriate controls.

### 6.1. Docking Score Is Not Enough

Molecular docking is widely used to predict ligand binding poses and prioritize compounds in structure-based virtual screening. Kitchen et al. reviewed the main concepts of docking and scoring and emphasized that docking depends on both conformational sampling and scoring-function evaluation [80]. This distinction is important for AI-generated molecules because a poor docking result may reflect either failure to sample a reasonable pose or failure of the scoring function to rank the correct pose. For generated molecules, docking can serve as an orthogonal filter after ligand-based or predictor-guided generation, but it should not be interpreted as a direct measurement of binding affinity.

The limitations of docking scores have been shown in systematic benchmarks. Warren et al. evaluated 10 docking programs and 37 scoring functions across multiple protein targets and found that performance varied across pose prediction, virtual screening, and affinity-ranking tasks [81]. This helps explain why a single docking score is insufficient for candidate selection. Docking programs remain valuable because they are fast and scalable. AutoDock Vina, for example, introduced a widely used scoring and search framework that improved speed and binding-mode prediction compared with earlier AutoDock implementations [45]. Speed, however, should not be confused with reliability.

For AI-generated molecules, docking is most informative when used comparatively. Generated molecules should be compared with known ligands, redocked crystallographic ligands, decoys, or molecules produced by an unoptimized prior model. Score distributions are more informative than isolated top scores, especially when a large generated library can produce apparently favorable scores by chance. The docking protocol should also be described clearly, including the receptor structure, binding-site definition, ligand and protein preparation, protonation-state treatment, grid settings, docking mode, number of poses, and whether redocking or control docking was performed.

### 6.2. Binding Pose and Key Interaction Analysis

Binding-pose inspection is often more informative than the docking score itself. A generated molecule should be examined to determine whether it occupies the intended binding site, avoids severe steric clashes, adopts a reasonable conformation, and forms chemically interpretable interactions. Here, a steric clash refers to an unrealistically close contact between ligand and protein atoms, or within the ligand itself, that would be energetically unfavorable and often indicates an implausible pose. For targets with established ligand-recognition patterns, pose inspection can help distinguish plausible candidates from molecules that receive favorable scores for unclear or artifactual reasons.

Key interaction analysis may include hydrogen bonds, salt bridges, aromatic interactions, hydrophobic contacts, metal coordination, hinge-binding interactions, or contacts with catalytic and allosteric residues. The relevant interactions depend on the target and binding mode. Adenosine triphosphate (ATP)-competitive kinase inhibitors, for example, are often assessed by hinge-region interactions, whereas allosteric ligands may require different structural criteria. The goal is not to force every generated molecule to reproduce known ligand interactions exactly, but to determine whether the proposed binding mode is chemically and mechanistically plausible.

Interaction analysis should also account for geometry and local context. A reported hydrogen bond is meaningful only when the donor–acceptor distance, angle, protonation state, and local environment are reasonable. Hydrophobic contacts and aromatic interactions should likewise be interpreted with attention to pocket complementarity and ligand strain. Merely listing interacting residues can create a false sense of structural support if the pose is distorted, poorly positioned, or inconsistent with known binding-site behavior. Recent work on PoseBusters reinforces this point by showing that predicted ligand poses should be evaluated for physical plausibility rather than only by score or root-mean-square deviation (RMSD) [30].

### 6.3. Rescoring and Binding Free-Energy Estimation

Docking scores are often followed by rescoring to improve candidate prioritization. Rescoring may involve consensus scoring, empirical or physics-based scoring functions, interaction fingerprints, molecular mechanics/Poisson–Boltzmann surface area (MM/PBSA), MM/GBSA, or more rigorous free-energy methods. For AI-generated molecules, rescoring can reduce dependence on a single docking score and provide an additional ranking layer before more expensive simulations or experiments.

MM/PBSA and MM/GBSA are widely used end-point methods for estimating relative binding free energies from molecular mechanics energies and implicit-solvent models. Genheden and Ryde reviewed these methods and emphasized the importance of calibration, testing, and validation when they are used for ligand-binding affinity estimation [13]. Their review is particularly relevant to generated molecules because MM/GBSA is often used as a post-docking filter, yet its numerical values are sensitive to input poses, force fields, dielectric settings, sampling protocols, and entropy treatment.

MM/GBSA values should therefore be interpreted with caution. They are often more useful for relative prioritization within a related compound set than for absolute prediction of experimental binding affinity. Small differences in calculated binding energy should not be overinterpreted unless uncertainty and protocol performance have been considered. More rigorous alchemical free-energy methods can provide stronger thermodynamic estimates in suitable settings, but they require careful system preparation, sufficient sampling, and substantially greater computational cost. These methods estimate binding free-energy differences by simulating a thermodynamic transformation between related molecular states. As reviewed by Mobley and Gilson, binding free-energy prediction remains an area in which progress depends strongly on benchmarking and protocol quality [82]. For practical evaluation, the main point is that free-energy calculations should match the stage of the study. Approximate methods can support triage, whereas more rigorous methods are better reserved for smaller and well-defined candidate series.

### 6.4. Molecular Dynamics and Interaction Persistence

Molecular dynamics simulation provides a way to examine whether a predicted protein–ligand complex remains stable under a specified force field and simulation conditions. In the evaluation of AI-generated molecules, MD can test whether a docked pose rapidly leaves the pocket, whether key interactions persist, whether the ligand adopts an unstable conformation, and whether the binding site remains compatible with the ligand over time.

MD is useful because it introduces protein and ligand flexibility, which docking often treats only approximately. De Vivo et al. reviewed the role of MD and related methods in drug discovery and discussed how these approaches can be used to study ligand binding, conformational change, allostery, and free-energy-related properties [83]. For generated molecules, this flexibility matters because a candidate that appears plausible in a rigid docking pose may behave differently once the protein, ligand, solvent, and ions are allowed to move.

Common MD-derived descriptors include protein RMSD, ligand RMSD, binding-site RMSD, root-mean-square fluctuation (RMSF), hydrogen-bond occupancy, contact persistence, radius of gyration, solvent-accessible surface area, and distances between ligand atoms and key residues. These descriptors can provide useful evidence about pose stability and interaction persistence. When combined with appropriate end-point or alchemical free-energy methods, MD-based sampling can also support affinity-oriented prioritization for a limited number of prioritized compounds.

MD results should still be interpreted according to the level of evidence they provide. Simulation outcomes depend on the starting pose, protein preparation, protonation states, ligand parameters, water placement, force field, simulation length, and analysis protocol. Short simulations can identify unstable poses or support a plausibility argument, but they usually cannot capture slow binding-site rearrangements, rare unbinding events, long-timescale induced-fit changes, or true residence time. MD-based analyses should therefore be distinguished from experimental validation and described according to the level of evidence they provide. Short trajectory stability or repeated contacts alone should not be interpreted as evidence of potency, whereas MD-based free-energy calculations can support binding-affinity assessment when the protocol, sampling, and validation are appropriate.

For AI-generated molecules, MD is best used selectively. Simulating thousands of generated molecules is usually impractical. A reasonable workflow applies MD only to a small set of candidates that have already passed molecular correctness assessment, medicinal chemistry filtering, target-aware scoring, docking, and pose inspection. This staged use of MD reduces computational cost and avoids overanalyzing molecules that are unlikely to be experimentally useful.

### 6.5. When Structure-Based Evaluation Becomes Misleading

Structure-based assessment can strengthen the evaluation of AI-generated molecules, but it can also create misleading confidence when used uncritically. A common problem is overreliance on a single receptor conformation. Many proteins are flexible, and ligand binding may depend on side-chain rearrangements, loop motion, induced fit, water networks, or allosteric states. Ensemble docking was developed partly to address this issue by docking ligands against multiple receptor conformations rather than a single rigid structure [84]. This approach does not remove uncertainty, but it can reduce the risk of drawing conclusions from one receptor model.

A second problem is circular evaluation. If molecules are generated or optimized using docking score as the reward, reporting docking score again as the main validation provides limited independent evidence. Orthogonal evaluation is especially important in such cases. Molecules optimized by docking should be checked using medicinal chemistry filters, pose geometry, rescoring, MD, alternative scoring functions, or experimental assays. Molecules optimized by an affinity predictor should ideally be evaluated by structure-based methods that were not used during optimization.

A third problem is selective reporting. Showing only the most visually convincing poses or the top-scoring molecules can exaggerate the apparent success of a generative model. A more transparent report should state how many generated molecules were prepared, how many were successfully docked, how many failed because of geometry or preparation issues, how many passed score thresholds, how many showed interpretable poses, and how many remained after visual inspection or rescoring. Failure rates matter because they show whether a generator consistently produces structurally plausible molecules or only a small number of attractive examples.

Structure-based evaluation should also remain connected to medicinal chemistry and experimental feasibility. A molecule may dock well but contain reactive groups, poor solubility, excessive lipophilicity, high synthetic complexity, or a motif associated with assay interference. Conversely, a molecule with a moderate docking score but clear synthetic accessibility and reasonable structure–activity relationship potential may be more useful as an experimental starting point. Practical evaluation should integrate structure-based evidence with chemical feasibility and testability.

### 6.6. Practical Reporting of Structure-Based Assessment

For structure-based evaluation to be useful, studies need to report enough methodological detail for readers to interpret and reproduce the results. Docking-based studies should at least specify the receptor structure, binding-site definition, ligand and protein preparation, protonation-state treatment, docking software, scoring function, search settings, number of poses, and candidate-selection criteria. When possible, redocking of a crystallographic ligand or docking of known actives and decoys can provide a useful reference for interpreting docking performance.

Candidate-level reporting should include both scores and poses. Representative molecules should be shown with binding poses, key interactions, and relevant molecular properties. If MM/GBSA or another rescoring method is used, the protocol should describe the input structures, minimization or sampling procedure, dielectric settings when relevant, and whether entropy was considered. If MD or MD-based free-energy estimation is used, reporting should include simulation length, force field, ligand parameterization, solvent model, equilibration protocol, analysis windows, and definitions of hydrogen bonds or contacts, and the free-energy protocol where applicable.

The language used to describe structure-based results should remain matched to the evidence. Without experimental evidence, molecules should be described as docked candidates, computationally prioritized compounds, predicted binders, or structurally plausible candidates, rather than confirmed inhibitors or active compounds. This distinction is important because structure-based methods can support prioritization, but they do not establish biological activity.

## 7. Translational Readiness: From Generated Molecules to Testable Candidates

AI-generated molecules are often evaluated as computational outputs, but drug discovery ultimately depends on compounds that can be synthesized, obtained, tested, and optimized. A molecule may have favorable generative metrics, acceptable drug-like properties, a high predicted activity score, and a plausible docking pose, yet still be unsuitable for follow-up if it is difficult to synthesize, commercially unavailable, affected by clear ADMET liabilities, incompatible with the intended assay, or too close to previously disclosed chemical space. Translational readiness therefore asks whether a generated molecule is realistic enough to justify experimental investment.

This layer is not a substitute for experimental validation. It provides a final plausibility check before generated molecules are described as practical candidates. In early discovery, only a small fraction of computationally proposed structures can usually be synthesized or tested. A practical translational assessment should therefore consider not only whether molecules look promising in silico, but also whether they are experimentally actionable.

### 7.1. Synthesizability, Purchasability, and Route Availability

Although the SA score provides a useful first-pass estimate of synthetic difficulty, candidate-level prioritization requires stronger evidence. A molecule with a favorable SA score may still be difficult to prepare if it requires unavailable starting materials, unstable intermediates, difficult stereochemical control, or low-yielding transformations. In contrast, a molecule with a less favorable score may be accessible if a known route exists or close analogues have already been synthesized. For a generated molecule to be experimentally actionable, researchers need evidence that it can be purchased, synthesized through a reasonable route, or assembled from accessible building blocks.

Computer-assisted synthesis planning can provide a more informative route-level assessment. Retrosynthesis tools and reaction-prediction models can help identify plausible disconnections, search known reaction precedents, and estimate whether proposed transformations are chemically reasonable [58,85,86]. Such tools are useful because they shift the evaluation from whether a molecule appears synthetically simple to whether a plausible route may exist in known chemistry. Predicted routes still require chemical review, because real synthesis depends on reaction conditions, selectivity, purification, reagent quality, cost, scale, and laboratory constraints.

Synthetic readiness can be viewed at three practical levels. The first is approximate tractability, supported by SA score or related descriptors. The second is route plausibility, supported by retrosynthetic analysis, building-block availability, or known reaction precedents. The third is practical accessibility, supported by commercial availability, existing analogue synthesis, or an expert-reviewed route. For molecules proposed as final candidates, reporting should move beyond a single synthetic-accessibility score and provide candidate-level evidence whenever possible.

### 7.2. ADMET and Developability-Oriented Filtering

ADMET properties matter because early target activity is not enough for drug discovery. Compounds with poor solubility, permeability, metabolic stability, transporter-related liabilities, drug–drug interaction risk, or toxicity liabilities may fail despite favorable potency. Waring et al. analyzed attrition data from four major pharmaceutical companies and reported that physicochemical properties were associated with candidate quality and the risk of safety-related clinical failure [87]. This does not mean that in silico ADMET predictions can determine clinical success, but it supports the inclusion of developability-oriented filtering before generated molecules are selected for follow-up.

For AI-generated molecules, in silico ADMET evaluation is most useful as risk screening. SwissADME provides rapid predictions of physicochemical properties, pharmacokinetics, drug-likeness, and medicinal chemistry friendliness for small molecules [14]. ADMETlab 2.0 integrates a broad set of ADMET-related endpoints, including physicochemical properties, medicinal chemistry descriptors, ADME, toxicity, and toxicophore predictions [15]. More recently, ADMETlab 3.0 expanded the platform with updated datasets, models, and endpoints for ADMET prediction [88]. Related generative studies have also begun to incorporate toxicity-oriented objectives into molecular generation, illustrating that developability considerations can be used not only as post hoc filters but also as design constraints during molecular generation [89]. These tools and related studies show how early developability checks can be incorporated into computational evaluation workflows.

ADMET predictions should not be treated as final evidence of safety or pharmacokinetic success. These models are trained on heterogeneous datasets that may differ in assay format, species, endpoint definition, and data quality. Their reliability may also decline for generated molecules that lie far from the chemical space represented in the training data. ADMET results are best interpreted as risk annotations that help prioritize molecules, not as definitive statements that a compound is safe, bioavailable, or developable.

ADMET assessment should be selective and context-dependent. Depending on the project, relevant endpoints may include aqueous solubility, gastrointestinal absorption, blood–brain barrier permeability, Cytochrome P450 (CYP) inhibition, P-glycoprotein substrate or inhibitor probability, human ether-à-go-go-related gene (hERG) liability, hepatotoxicity, mutagenicity, and general toxicity alerts. The selected endpoints should match the therapeutic context, route of administration, and intended assay system. Very strict filters applied too early may remove optimizable hits, whereas ignoring ADMET until the final stage can lead to unrealistic candidate selection. A balanced approach is to remove molecules with severe or obvious liabilities early, while using softer thresholds or risk annotations for properties that may be improved later.

### 7.3. Patent-Context Novelty and Patent-Overlap Screening

Novelty in molecular generation is often measured against the training set, but translational novelty is broader. A generated molecule may be absent from the training set but already disclosed in patents, present as a close analogue in a known series, or covered by broad Markush claims. If the goal is to identify experimentally actionable or potentially protectable candidates, exact novelty against the training set is not sufficient.

Patent-derived chemical resources can help assess whether generated molecules overlap with known disclosed chemical space. SureChEMBL is a large-scale patent document database that extracts chemical structures from life-science patent literature through text and image mining [90]. More recently, Gadiya et al. examined SureChEMBL from a drug discovery perspective and analyzed the medicinal chemistry relevance of patent-derived chemical data [91]. These resources allow researchers to examine whether generated molecules are exact matches or close analogues of compounds disclosed in patents.

For AI-generated molecules, a basic patent-context analysis may include exact matching against patent-derived databases, similarity searches against patented compounds, and scaffold-level comparisons with disclosed chemical series. These analyses still need careful wording. Exact absence from a patent database does not establish freedom to operate. Patent claims may cover broad chemical classes, substituent patterns, salts, stereoisomers, uses, formulations, or synthetic routes. A proper freedom-to-operate assessment requires legal expertise and jurisdiction-specific analysis. In a scientific article, such analysis is better described as patent-context novelty screening or patent-overlap analysis, not as proof of freedom to operate.

Patent-context novelty remains useful even when formal intellectual-property claims are not the main objective. It can help distinguish genuine chemical exploration from rediscovery of heavily disclosed compound series. It can also help prioritize generated molecules that are less likely to be trivial analogues of known patented chemistry. This consideration should still be balanced against biological plausibility and medicinal chemistry quality. A molecule far from patent space is not necessarily useful if it lacks target relevance, synthetic feasibility, or experimental testability.

### 7.4. Experimental Prioritization and Candidate-Level Rationale

The final purpose of translational readiness evaluation is to produce a small, rationally selected candidate set for experimental testing. This step should integrate all previous evidence, including molecular correctness, medicinal chemistry feasibility, novelty, target relevance, structure-based plausibility, synthetic accessibility, ADMET risk, assay compatibility, and patent-context novelty. No single score should dominate final selection unless the study has a clearly justified objective.

For final prioritized molecules, candidate-level rationale is more informative than aggregate scores alone. Authors should explain why each molecule was retained, what evidence supports its selection, and what uncertainties remain. Useful information may summarize the molecular structure, predicted activity or target-aware score, novelty and patent-context information, structural-alert status, purchasability or route plausibility, selected ADMET risks, assay compatibility, and structure-based evidence if used.

Experimental prioritization should also distinguish computational candidates from experimentally validated hits. Without synthesis, purchase, or biological testing, molecules should be described as computationally prioritized, experimentally actionable, or proposed for follow-up rather than as confirmed hits, inhibitors, or leads. When experiments are performed, reporting negative or inactive tested compounds is also valuable because it clarifies the selection process and helps future studies identify where computational prioritization succeeded or failed.

## 8. Common Failure Modes in Evaluating AI-Generated Molecules

**Figure 2:**
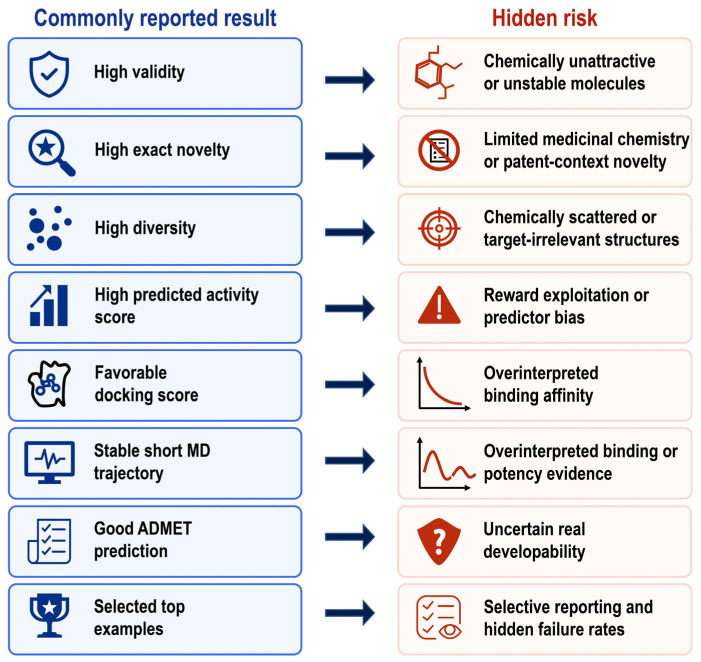
Schematic of common failure modes in the evaluation of AI-generated molecules. For each commonly reported evaluation result (**left**), a corresponding hidden risk (**right**) may lead to overinterpretation if not checked by complementary evidence. The full list of failure modes and recommended safeguards is given in Table 2.

Commonly reported evaluation results can give an incomplete view of AI-generated molecules when they are interpreted beyond the level of evidence they provide. A valid structure may still be chemically unattractive, an exact-novel molecule may remain close to known or patented chemistry, a diverse set may be unrelated to the intended target, and a high surrogate score may reflect reward exploitation or predictor bias rather than biological relevance. Structure-based and translational evaluations introduce additional risks when docking scores, selected poses, short simulations, SA scores, or in silico ADMET predictions are treated as stronger evidence than they are. Figure 2 summarizes these recurring mismatches between reported evidence and hidden risks, while Table 2 also lists the corresponding safeguards.

At the level of basic generative metrics, the most frequent failures arise from interpreting formal molecular quality as practical usefulness. Validity, uniqueness, novelty, and diversity are necessary for model evaluation, but they do not by themselves establish medicinal chemistry value. False validity occurs when parsable molecules contain unstable motifs, undesirable substructures, or poor physicochemical properties. False novelty arises when absence from the training set is treated as meaningful chemical innovation, even though the molecule may share a scaffold or close analogue relationship with known compounds. Diversity inflation happens when broad chemical dispersion is achieved at the cost of target relevance, synthetic tractability, or property balance. These risks are best addressed by reporting not only raw generative metrics, but also scaffold-level analyses, nearest-neighbor similarity, property distributions, and diversity after key filtering steps.

Target-aware generation introduces a second class of failures. Activity predictors, DTI or DTA models, docking scores, and composite rewards are often useful for prioritization, but they can also guide models toward scoring artifacts. Reward exploitation occurs when optimization improves the surrogate score without improving the underlying medicinal chemistry or biological plausibility [75,76,77,78]. Predictor bias exists when highly scored generated molecules fall outside the chemical or descriptor space in which the model was validated. These problems are particularly important when the same model is used both to optimize and to evaluate generated molecules. Appropriate safeguards include external or scaffold-based validation, applicability-domain or uncertainty analysis, and orthogonal evaluation using methods that were not part of the reward function.

Structure-based assessment can also be overinterpreted. Docking scores are useful for large-scale prioritization, but isolated top scores do not demonstrate binding or potency. This problem is amplified when score distributions, failed docking attempts, receptor preparation choices, and reference compounds are not reported. Recent analyses have also shown that docking scores can be influenced by molecular size, supporting the need for size-aware controls, realistic baselines, and pose-level inspection rather than ranking by raw score alone [48]. Rescoring and molecular dynamics can strengthen structure-based assessment, but they remain protocol-dependent. MM/GBSA is often most appropriate for relative prioritization within a filtered set, while MD-based free-energy calculations can support affinity-oriented assessment for a limited number of prioritized compounds when properly validated. However, short MD trajectory stability or contact persistence alone should not be interpreted as evidence of potency. When these methods are used, simulation conditions, force fields, ligand parameters, analysis windows, definitions of contacts or hydrogen bonds, and the free-energy protocol, where applicable, should be reported.

At the translational layer, computationally attractive molecules may still fail because they are difficult to synthesize, unavailable for testing, affected by obvious ADMET liabilities, or unsuitable for the intended assay. A favorable SA score should therefore be described as predicted synthetic tractability rather than proof of practical synthesis. Similarly, ADMET predictions should be presented as risk annotations, not as evidence of pharmacokinetic or safety success. Weak reporting can compound all of these problems. Selective presentation of only the best-scoring molecules, most attractive poses, or most favorable simulations may obscure high failure rates in the raw generated library. More transparent studies should report the evaluation funnel, including how many molecules were generated, standardized, retained after medicinal chemistry filters, considered target-relevant, successfully docked or structurally assessed, and finally selected for synthesis or testing.

These failure modes share a common source. One layer of computational evidence is often interpreted as stronger than it actually is. Recognizing these distinctions does not reduce the value of AI-driven molecular generation. Rather, it clarifies how generated molecules should be interpreted. Failure-mode analysis therefore provides the diagnostic basis for the staged evaluation framework proposed in the next section.

## 9. A Practical Staged Evaluation Framework

The evaluation of AI-generated molecules is better organized as a staged process than reduced to a single score or a small set of disconnected metrics. Each stage addresses a different level of evidence, from basic molecular correctness to experimental actionability.

This distinction is important because success in molecular generation does not necessarily mean success in drug discovery. A generator may perform well as a model while producing few molecules that are suitable for experimental follow-up. Conversely, a more modest model may still be useful if its molecules can pass chemical, biological, structural, and translational filters. This staged logic reflects the usual workflow of early drug discovery. Large generated compound sets are first screened with simple, inexpensive, and broadly applicable criteria, and are then refined using more specific, target-dependent, and resource-intensive analyses. The framework proposed here is not intended as a rigid universal standard. It instead provides a practical structure for aligning the strength of computational claims with the evidence used to support them.

### 9.1. Aligning Evaluation Depth with Study Type

The depth of evaluation should depend on the purpose of the study. A benchmark study may focus mainly on validity, uniqueness, novelty, diversity, and distributional similarity, as in GuacaMol and MOSES [1,2]. A target-aware method paper should extend the evaluation to target-relevance scoring, predictor reliability, applicability-domain or uncertainty analysis, and, when appropriate, structure-based plausibility. A prospective discovery study requires stronger candidate-level evidence, including synthetic or commercial accessibility, ADMET risk assessment, assay compatibility, structural rationale where relevant, and experimental testing when possible. These study types should not be judged by identical requirements, because they support different levels of scientific claim.

This distinction is important because the same generated molecule can substantiate different claims depending on the evidence available. If only basic generative metrics are reported, the claim should be limited to model-level behavior. If medicinal chemistry, target-aware, and structure-based analyses are added, the study can support computational prioritization. Stronger claims about active compounds, hits, inhibitors, or candidates require experimental evidence. The goal of staged reporting is therefore not to increase reporting burden uniformly, but to make the evidentiary basis of each claim explicit.

### 9.2. Six Stages and Minimum Reporting Items

The six stages shown in Figure 1 are ordered by increasing specificity, evidentiary burden, and resource cost. Each paragraph below states what kind of evidence a stage provides and the boundary of the claim that this evidence can support; the corresponding minimum reporting items, organized by study type, are given in Table 3.

Molecular correctness is the entry layer. It establishes that generated outputs are chemically parsable and standardized, not that they are practical drug-discovery candidates. A parsable structure can still contain unstable motifs, undesirable substructures, or unfavorable properties. For three-dimensional generators, geometric and physical plausibility should also be confirmed before any structure-based step.

Medicinal chemistry feasibility provides early chemical triage by assessing whether molecules fall within a reasonable physicochemical and structural profile. This stage can identify obvious liabilities and prioritize molecules for further evaluation, but it does not confirm synthesizability, developability, or biological activity.

Novelty and diversity in context describe how a generated set relates to existing chemistry rather than whether it is valuable. Exact novelty against the training set, scaffold-level novelty, nearest-neighbor similarity, and diversity after filtering answer different questions and should not be conflated. For target-directed or prospective studies, novelty is most informative when interpreted against known active compounds, relevant chemical series, and patent-derived chemical space.

Target relevance and prediction reliability provide computational evidence that molecules may be relevant to a specific target, conditional on the scoring model being reliable in the chemical space being explored. Such evidence remains predictive rather than experimental. When the same model is used both to optimize and to evaluate generated molecules, reward exploitation becomes a particular concern, so orthogonal validation together with applicability-domain or uncertainty information is needed to interpret this stage.

Structure-based plausibility offers geometric and energetic evidence that a molecule may be compatible with a binding site, when a reliable target structure or pocket model is available. Docking, pose and interaction analysis, rescoring, MM/GBSA, and MD-based free-energy calculations can support structural or affinity-oriented prioritization, but their outputs depend on protocol, sampling, and controls. They should therefore not be treated as substitutes for biochemical or biophysical validation.

Translational and experimental readiness asks whether prioritized molecules can realistically enter follow-up studies. The evidence required scales with the claim: predicted synthetic-accessibility and ADMET values are risk annotations rather than proof of synthesis or safety, whereas proposed experimental candidates require stronger route-level, availability, and assay-readiness evidence.

Table 3 summarizes minimum recommended reporting items for three common types of AI molecular generation studies. A more detailed stage-by-stage checklist is provided in Table S2.

### 9.3. Software Support and Practical Reference Values

At present, no single generally accepted software package can reliably evaluate all stages of AI-generated molecules at the same time. The reason is that the six stages ask different questions. Some stages require cheminformatics standardization. Some require property or ADMET prediction. Others require target-aware models, protein structures, synthesis planning, or patent-context searches. A staged evaluation therefore usually needs a combination of tools rather than one universal platform.

Software should be selected according to the evaluation purpose and the claim being made. More reliable use requires clear input standardization, documented algorithms, suitable reference compounds or baselines, and external validation where available. When predictive models are used, applicability-domain or uncertainty information should also be considered. Representative software tools and web-based platforms for each stage are summarized in Table S3. Practical reference values and interpretation limits are summarized in Table S4. These values are intended as context-dependent guidance rather than universal pass/fail thresholds.

### 9.4. Reporting the Evaluation Funnel and Candidate Rationale

A practical evaluation should report how molecules survive through the evaluation funnel. A clear reporting format may include the number of total generated molecules, valid molecules, unique molecules, molecules passing medicinal chemistry filters, molecules retained after novelty or diversity assessment, molecules considered target-relevant, molecules successfully docked or structurally assessed, and molecules finally selected for synthesis, purchase, or testing. This type of survival reporting is important because a model that yields a few attractive examples from a very large and mostly unusable raw set should not be interpreted in the same way as a model that consistently produces chemically feasible and target-relevant candidates.

Candidate-level reporting remains essential for final prioritized molecules. Authors should provide molecular structures, key physicochemical properties, novelty information, predicted activity or target-aware scores, uncertainty or applicability-domain information if available, structure-based evidence if used, synthetic-accessibility or purchasability evidence, and major ADMET or assay-interference warnings. Such reporting helps readers evaluate why each molecule was selected and where uncertainty remains. It also supports reproducibility and is consistent with the findable, accessible, interoperable, and reusable (FAIR) principle for scientific data management and stewardship [92].

### 9.5. Application of the Framework to Representative Examples

The staged framework can be applied to different types of molecular generation studies, but not all studies require the same depth of evaluation. To illustrate this point, Table S5 summarizes five representative examples across the six evaluation stages. GuacaMol and MOSES represent benchmark studies, where the main evidence concerns molecular correctness, uniqueness, novelty, diversity, and distributional similarity [1,2]. REINVENT represents reward-guided molecular generation, where target-aware scoring can guide optimization but also requires attention to reward exploitation and independent validation [3]. GENTRL-based DDR1 inhibitor discovery illustrates a prospective setting in which generated molecules were prioritized, synthesized, and experimentally tested [33]. Rentosertib, formerly ISM001-055, provides a translationally advanced example of AI-assisted molecular discovery, where evidence extends beyond computational prioritization to preclinical and clinical evaluation [41,42]. Pocket2Mol represents pocket-conditioned three-dimensional molecular generation, where geometric plausibility, pocket compatibility, and structure-based assessment become central [26].

These examples are not intended as a ranking of individual studies. They instead show how the framework can align evaluation depth with study purpose. Benchmark studies mainly support claims about model behavior. Target-aware method papers require additional evidence about predictor reliability and reward independence. Structure-based generation studies require geometric and pose-level assessment. Prospective discovery studies require candidate-level rationale and, where possible, experimental testing. A structured comparison of these examples is provided in Table S5.

## 10. Conclusions and Perspectives

AI-driven molecular generation has become a useful approach for exploring chemical space and proposing new molecular structures in early-stage drug discovery. The value of generated molecules, however, cannot be judged from generative metrics alone. Validity, uniqueness, novelty, and diversity describe basic model behavior, but they do not show whether a molecule is chemically credible, biologically relevant, structurally plausible, or experimentally actionable. Benchmarking platforms such as GuacaMol and MOSES have made molecular generation studies easier to compare, but their metrics are designed mainly for model evaluation rather than for direct confirmation of drug discovery value [1,2]. The contribution of this review is to organize established evaluation practices into a staged, failure-aware, and evidence-graded framework. This structure helps distinguish model-level success from candidate-level evidence and experimental validation.

Several methodological priorities follow from this view. Future studies should place greater emphasis on prediction reliability. When QSAR, DTI, or DTA models are used to score generated molecules, authors should report the training data, endpoint definition, external validation, and whether the generated molecules fall within the applicability domain of the model [5,73,74,93]. This issue is especially important because molecular generation is often used to explore chemical regions that are poorly represented in existing activity datasets. Orthogonal evaluation deserves similar attention. If a molecule is optimized using one surrogate score, that score should not also be used as independent validation. Independent checks should instead come from a different evidence layer. Recent pose-validation work such as PoseBusters has shown that apparently successful docking or AI-generated poses may still fail basic chemical and geometric plausibility checks, supporting the need for independent structural quality control [30]. Evaluation should also become more transparent and reproducible. Reporting how molecules survive through each stage of the evaluation funnel, together with candidate-level provenance, makes both successes and failures easier to interpret and aligns molecular generation studies with the FAIR principles for scientific data management [92].

Prospective validation remains the most informative form of evaluation, but it is still rare relative to the rapidly growing number of generative models published each year. Even small-scale synthesis and testing can provide valuable information about model behavior, scoring reliability, and failure modes. In the longer term, workflows that connect molecular generation with synthesis planning, experimental testing, and model updating are likely to be more informative than isolated generation-and-ranking pipelines. Such workflows will require careful reporting of failed as well as successful candidates, rather than only selected positive examples. AI-generated molecules should ultimately be treated as testable hypotheses rather than conclusions. Their evaluation should move beyond validity and novelty toward a broader assessment of chemical quality, target relevance, structural plausibility, and experimental actionability. A staged and transparent evaluation framework cannot guarantee discovery success, but it can reduce misleading claims, improve reproducibility, and help identify molecules that are more suitable for follow-up in real drug discovery settings.

## Figures and Tables

**Table 1:** Representative classes of molecular generative models and their evaluation-relevant features.

Model Family	Representative Example	Molecular Representation	Conditioning or Goal Direction	Evaluation-Relevant Note
Sequence model based on recurrent neural network (RNN) or Transformer	REINVENT [3]; ReLeaSE [37]	SMILES or SELFIES	RL with a user-defined reward	Reward exploitation remains a risk; SELFIES provides validity by construction.
Variational autoencoder	JT-VAE [22]; GENTRL [33]	Graph fragments and latent space	Property guidance in latent space	Validation often relies on latent interpolation; novelty is defined relative to the training set.
Generative adversarial network	MolGAN [23]	2D graph	Property-conditioned discriminator	Mode collapse and limited scaffold diversity are common concerns.
Autoregressive graph model	GCPN [24]	2D graph	RL with a stepwise property reward	Validity depends on valence-aware construction rules.
Graph normalizing flow	GraphAF [25]; MoFlow [34]	2D graph	Exact likelihood estimation through invertible transformations	Validity depends on construction rules; likelihood is exact, but training stability can be a concern.
3D autoregressive model	Pocket2Mol [26]	3D atomic graph	Pocket-conditioned generation	3D pose validity is required; PoseBusters-type checks are relevant.
3D equivariant diffusion model	TargetDiff [27]; DiffSBDD [28]; EDM [35]; DiffMC-Gen [36]	3D coordinates	Pocket-conditioned denoising	Score distributions and pose geometry should be reported, rather than only top-scoring examples.
3D language-geometry hybrid model	Lingo3DMol [29]	Fragment SMILES and local coordinates	Pocket and non-covalent interaction guidance	Both topological and geometric validity need to be assessed.

**Table 2:** Common failure modes and recommended safeguards in evaluating AI-generated molecules.

Failure Mode	Typical Manifestation	Why It Matters	Recommended Safeguard
False validity	Molecules are parsable but chemically unattractive or unstable	Validity does not guarantee medicinal chemistry value	Combine sanitization with property filters and expert inspection
False novelty	Molecules are absent from the training data but remain close to known analogues	Exact novelty may overstate chemical innovation	Report scaffold novelty, nearest-neighbor similarity, and patent-context novelty
Diversity inflation	High diversity reflects scattered or irrelevant chemistry	Diversity alone does not indicate useful exploration	Report diversity among filtered and target-relevant molecules
Reward exploitation	Molecules achieve high reward by exploiting scoring artifacts	Optimization may improve the score rather than the molecule	Use hard constraints, orthogonal validation, and score-component reporting
Predictor bias	High-scoring molecules fall outside the reliable domain of the predictor	Surrogate predictions may extrapolate poorly	Include applicability-domain and uncertainty analyses
Docking overinterpretation	Docking scores are treated as binding affinity	Docking scores are approximate and system-dependent	Combine score distributions with pose inspection and control experiments or reference compounds
MM/GBSA overinterpretation	Small energy differences are treated as decisive	End-point free-energy estimates are protocol-sensitive	Use MM/GBSA mainly for relative prioritization and report protocol details
MD overinterpretation	Short trajectory stability is treated as proof of potency	MD results are protocol- and sampling-dependent	Separate stability analysis from MD-based free-energy estimation
Synthetic infeasibility	Top candidates are difficult to synthesize or obtain	Testability is required for practical discovery	Check SA score, purchasability, retrosynthesis, and building-block availability
ADMET neglect	High-scoring molecules have obvious developability risks	Target activity alone is insufficient	Include selected ADMET endpoints relevant to the project
Weak reporting	Only top examples or aggregate scores are shown	Reproducibility and interpretation become difficult	Report filtering rules, survival rates, thresholds, and the rationale for final candidate selection

**Table 3:** Minimum recommended reporting items for three types of AI molecular generation studies.

Evaluation Stage	Benchmark Study	Target-Aware Method Paper	Prospective Discovery Study
Molecular correctness	Report molecular representation, parsing toolkit and version, standardization rules, validity, uniqueness, duplicate removal, and sample size.	Report all benchmark-level items, together with target-conditioned generation success or failure rates where relevant. For 3D methods, include basic geometric and pose-validity checks.	Report all target-aware items and provide candidate-level structures with clear salt, charge, tautomer, stereochemical, and standardization status.
Medicinal chemistry feasibility	Report property distributions and basic drug-likeness or lead-likeness descriptors, including MW, cLogP, TPSA, HBD/HBA, rotatable bonds, QED, SA score, and structural alerts.	Report property distributions before and after target-aware optimization or filtering. Describe how medicinal chemistry constraints were used in reward design, filtering, or candidate selection.	Report candidate-level physicochemical properties, structural alerts, synthetic-accessibility evidence, purchasability or route plausibility, and the rationale for retaining or excluding flagged compounds.
Novelty and diversity in context	Define the reference set and report exact novelty, scaffold novelty, fingerprint and similarity metric, pairwise or internal diversity, and distributional comparisons where appropriate.	Report novelty and diversity relative to the training set, known active ligands, target-relevant chemical space, and molecules generated by unoptimized baselines.	Report novelty relative to known actives, disclosed analogues, patent-derived chemical space where relevant, and commercially available or synthesized analogues.
Target relevance and prediction reliability	Usually not required unless the benchmark is target-specific. If target-specific, report the target, endpoint, scoring rule, and reference compounds.	Report the activity, DTI, DTA, pharmacophore, similarity, docking, or composite scoring model; endpoint definition; training data; validation strategy; threshold selection; and applicability-domain or uncertainty information where available.	Report candidate-level target-relevance evidence, preferably including orthogonal computational evaluation and experimental assay results when available. If predictions are used, indicate whether final candidates fall within the reliable prediction domain.
Structure-based plausibility	Optional unless the benchmark is structure-based. When used, report receptor structure, docking protocol, scoring function, score distributions, and reference ligands or baselines.	For structure-based or pocket-conditioned generation, report docking success rates, score distributions, pose inspection criteria, geometric checks, key interactions, size-aware controls, and rescoring, MD, or MD-based free-energy protocols if used.	Provide candidate-level poses, interaction rationale, comparison with known ligands, and rescoring, MD, or MD-based free-energy evidence if used. If affinity estimates are claimed, report the free-energy method, sampling protocol, and validation strategy.
Translational and experimental readiness	Usually limited to basic chemistry filters and optional synthetic-accessibility estimates.	Report whether generated molecules are synthetically plausible, commercially available, or suitable for future testing when specific candidates are emphasized. Include selected ADMET or assay-risk annotations where relevant.	Report final candidate structures, synthetic or purchasability evidence, route or building-block information where available, selected ADMET risks, assay compatibility, patent-context novelty if used, tested compounds, negative results where available, and final selection rationale.

## Data Availability

No new data were created or analyzed in this study. Data sharing is not applicable to this article.

## Supplementary Materials

The following supporting information can be downloaded at: https://www.mdpi.com/article/10.3390/ijms27135916/s1.

## Abbreviations

The following abbreviations are used in this manuscript:

ADMET	Absorption, distribution, metabolism, excretion, and toxicity
AI	Artificial intelligence
ATP	Adenosine triphosphate
cLogP	Calculated logarithm of the octanol/water partition coefficient
CYP	Cytochrome P450
DDR1	Discoidin domain receptor 1
DTA	Drug–target affinity
DTI	Drug–target interaction
EDM	Equivariant diffusion model
FAIR	Findable, accessible, interoperable, and reusable
FCD	Fréchet ChemNet Distance
Fsp3	Fraction of sp3-hybridized carbon atoms
HBA	Hydrogen-bond acceptor
HBD	Hydrogen-bond donor
hERG	Human ether-à-go-go-related gene
IntDiv	Internal diversity
MD	Molecular dynamics
MM/GBSA	Molecular mechanics/generalized Born surface area
MM/PBSA	Molecular mechanics/Poisson–Boltzmann surface area
MW	Molecular weight
PAINS	Pan-assay interference compounds
QED	Quantitative estimate of drug-likeness
QSAR	Quantitative structure–activity relationship
RL	Reinforcement learning
RMSD	Root-mean-square deviation
RMSF	Root-mean-square fluctuation
RNN	Recurrent neural network
SA	Synthetic accessibility
SE(3)	Special Euclidean group in three dimensions
SELFIES	Self-referencing embedded strings
SMILES	Simplified molecular-input line-entry system
TNIK	TRAF2- and NCK-interacting kinase
TPSA	Topological polar surface area
VAE	Variational autoencoder
