# Supplementary material for: Evaluating AI-Generated Molecules for Drug Discovery: From Generic Metrics to Translational Readiness
Source: Int J Mol Sci. 2026 Jun 30;27(13):5916. doi: 10.3390/ijms27135916 (PMC13362286; doi:10.3390/ijms27135916)
Supplement: Supplementary file 1 [file ijms-27-05916-s001.zip › ijms-4377961-supplementary.pdf]

Supporting Information

Evaluating AI-Generated Molecules for Drug Discovery: From Generic Metrics to Translational Readiness

Xiaomeng Liu and Huanxiang Liu *

Centre for Artificial Intelligence Driven Drug Discovery, Faculty of Applied Sciences, Macao Polytechnic University, Rua de Luís Gonzaga Gomes, Macau SAR, China

* Correspondence: hxliu@mpu.edu.mo

Supplementary Tables

Table S1. Common metrics for evaluating AI-generated molecules and their interpretation limits.

Evaluation Aspect	Common Metric or Analysis	What It Measures	What It Does Not Establish	Common Pitfall
Molecular validity	Valid SMILES ratio; RDKit sanitization; PoseBusters-style geometric checks for 3D	Whether generated strings can be parsed into chemically valid molecular graphs, or whether 3D poses satisfy basic geometric and physical constraints	Drug-likeness, stability, synthesizability, or biological relevance	Treating chemical parsability as evidence of practical usefulness
Redundancy control	Uniqueness; duplicate removal after canonicalization	Whether the model repeatedly generates identical molecules	Chemical diversity or scaffold innovation	Reporting high uniqueness without assessing the quality of chemical-space exploration
Exact novelty	Fraction absent from the training set or reference set	Whether generated molecules are exact new structures relative to a defined dataset	Scaffold novelty, analogue-level novelty, or patent-context novelty	Equating exact novelty with meaningful chemical novelty
Scaffold novelty	Bemis–Murcko scaffold novelty; scaffold frequency	Whether generated compounds explore new core frameworks	Whether the scaffolds are synthetically accessible or target-relevant	Overvaluing rare scaffolds without medicinal chemistry inspection
Molecular diversity	Pairwise Tanimoto distance; scaffold diversity; chemical-space dispersion	The breadth of the generated chemical space	Relevance to the target or disease context	Inflating diversity by generating chemically irrelevant structures
Drug-likeness	QED; Lipinski rule of five; Veber rules; physicochemical property ranges	Whether compounds fall within common drug-like property ranges	Target potency, selectivity, or ADMET safety	Using empirical rules as strict pass/fail criteria
Synthetic feasibility	SA score; retrosynthesis score; route availability; purchasability	Whether compounds are likely to be chemically tractable	Actual synthetic cost, yield, scalability, or route robustness	Assuming that an SA score alone confirms synthesizability

Structural alerts	PAINS filters; reactive-group filters; toxicophore alerts	Whether molecules contain undesirable or assay-interfering substructures	The full safety profile or context-dependent liability	Removing all flagged molecules without expert review
Target relevance	QSAR score; DTA/DTI prediction; activity classifier	Predicted biological relevance to a target	True biochemical potency or cellular activity	Treating surrogate predictions as experimental evidence
Ligand similarity	Similarity to known actives; matched molecular pairs; fragment recovery	Whether generated molecules retain target-associated chemical motifs	Novel binding modes or true activity	Allowing excessive similarity to produce trivial analogue generation
Pharmacophore fit	Pharmacophore matching; feature overlap	Whether molecules preserve key interaction hypotheses	Full binding geometry or conformational stability	Overconstraining generation toward known chemotypes
Docking evaluation	Docking score; docking enrichment; pose ranking	Approximate compatibility with a binding pocket	True binding affinity or residence time	Ranking molecules by docking score alone
Binding-mode plausibility	Pose inspection; hinge or contact analysis; interaction fingerprints	Whether binding poses are chemically interpretable	MD-derived stability or experimental potency	Reporting interactions without checking geometry and chemical context
Rescoring	MM/GBSA; consensus scoring; rescoring functions	Relative post-docking prioritization	Absolute binding free energy	Overinterpreting small score differences
MD-derived stability	MD RMSD; ligand RMSD; hydrogen-bond occupancy; contact persistence	Whether predicted complexes remain stable during simulation	Long-timescale binding or unbinding behavior, or true affinity	Using short MD simulations as definitive validation
ADMET feasibility	Solubility, permeability, CYP inhibition, hERG, and toxicity predictors	Early developability risks	Clinical safety or pharmacokinetic success	Treating in silico ADMET as final developability evidence
Translational novelty	Patent search; exact-patent filter; analogue-level novelty	Whether molecules avoid direct overlap with known or patented compounds	Formal freedom-to-operate status or jurisdiction-specific legal claim-space assessment	Treating exact-match patent novelty as sufficient intellectual property novelty
Experimental readiness	Purchasability; synthetic route; assay	Whether molecules can realistically enter experimental testing	Experimental success	Selecting computationally attractive molecules

	compatibility; prioritization score			that are difficult to test experimentally

Table S2. A practical staged checklist for evaluating AI-generated molecules.

Evaluation Stage	Main Question	Recommended Analyses	Minimum Reporting Items	Interpretation Caution
Molecular correctness	Can the generated output be interpreted as a valid and standardized molecule?	SMILES parsing, molecular graph construction, valence checking, sanitization, stereochemical handling, salt removal, and duplicate removal	Molecular representation, toolkit and version, standardization rules, validity rate, uniqueness rate, and duplicate-removal procedure	Validity confirms parsability and basic chemical representation, but not usefulness for drug discovery
Medicinal chemistry feasibility	Does the molecule have a reasonable property profile and avoid obvious chemical liabilities?	MW, cLogP, TPSA, HBD/HBA, rotatable bonds, QED, Lipinski and Veber rules, PAINS, reactive groups, SA score, and structural-alert filters	Property distributions, filtering thresholds, alert definitions, SA score distribution, and rationale for retaining or removing flagged molecules	Drug-likeness and lead-likeness rules are risk indicators rather than universal pass/fail criteria; SA score alone does not prove synthesizability
Novelty and diversity in context	Does the molecule expand chemical space in a meaningful and relevant way?	Exact novelty, scaffold novelty, Bemis–Murcko scaffold analysis, nearest-neighbor similarity, pairwise diversity, chemical-space visualization, and patent-context similarity when relevant	Reference dataset, novelty definition, scaffold method, fingerprint and similarity metric, diversity distribution, and patent-overlap search if used	Absence from the training set is not equivalent to scaffold novelty, medicinal chemistry novelty, patent-context novelty, or functional novelty
Target relevance and prediction reliability	Is the molecule related to the intended biological objective, and is the prediction reliable?	QSAR, DTI/DTA prediction, activity classifier, similarity to known actives, pharmacophore matching, fragment recovery, applicability-domain analysis, nearest-neighbor distance, uncertainty estimation, and calibration	Scoring model, endpoint definition, training data, validation performance, activity threshold, and applicability-domain or uncertainty information if available	Predicted activity is not experimental activity; novel molecules may lie outside the reliable prediction domain

Structure-based plausibility	Can the molecule adopt a plausible binding mode in the target structure?	Docking, pose inspection, interaction fingerprints, comparison with known ligands, rescoring, MM/GBSA, MD, RMSD/RMSF, contact persistence, and hydrogen-bond occupancy	Receptor structure, docking protocol, scoring function, score distributions, representative poses, MM/GBSA protocol if used, and MD force field, ligand parameters, simulation length, and analysis criteria if used	Docking score is not binding affinity; MM/GBSA is mainly useful for relative prioritization; short MD supports plausibility but does not prove binding
Translational and experimental readiness	Can the candidate realistically enter follow-up studies?	Purchasability, retrosynthetic route analysis, building-block availability, ADMET prediction, solubility, permeability, CYP, hERG, toxicity risk, assay compatibility, patent-context novelty, and final prioritization	Final candidate table, synthetic or purchasability evidence, selected ADMET endpoints, assay-interference warnings, patent-context analysis if used, selection rationale, and known limitations	Computational prioritization is not confirmed discovery; ADMET predictions are risk screening; patent-context novelty is not freedom to operate

Table S3. Representative software tools and web-based platforms for staged evaluation of AI-generated molecules.

Evaluation Stage	Evaluation Purpose	Representative Tools or Platforms	Typical Outputs	Interpretation Caution
Molecular correctness	Parse, standardize, sanitize, and check generated structures	RDKit, Open Babel, PoseBusters	Validity, canonical structures, stereochemical status, molecular descriptors, and 3D geometry or pose-quality checks	Results depend on toolkit settings, standardization rules, protonation states, stereochemical handling, and whether 3D checks are included.
Medicinal chemistry feasibility	Assess physicochemical properties, drug-likeness, lead-likeness, synthetic accessibility, and structural alerts	RDKit descriptors, SwissADME, ADMETlab, SA score implementations, PAINS and medicinal chemistry alert filters	MW, cLogP, TPSA, HBD/HBA, rotatable bonds, QED, SA score, and structural-alert flags	These outputs are early triage indicators. They do not prove synthesizability, safety, developability, or biological activity.

Novelty and diversity in context	Compare generated molecules with training data, known chemistry, active ligands, or patent-derived chemical space	RDKit fingerprints, Bemis–Murcko scaffold analysis, ChEMBL, ZINC, PubChem, SureChEMBL	Exact novelty, scaffold novelty, nearest-neighbor similarity, internal diversity, property distributions, and patent-context overlap	Novelty depends on the reference set and similarity definition. Exact novelty does not imply scaffold novelty, patent-context novelty, or freedom to operate.
Target relevance and prediction reliability	Estimate whether generated molecules are relevant to a target or biological endpoint	QSAR models, DTI or DTA models, DeepDTA-like models, GraphDTA-like models, MolTrans-like models, and pharmacophore tools such as LigandScout	Predicted activity or affinity, interaction probability, pharmacophore fit, similarity to known actives, and applicability-domain or uncertainty information where available	Predictions are only reliable within the model's validated domain. Independent validation is especially important when the same model is used for generation and evaluation.
Structure-based plausibility	Assess compatibility with a binding site and evaluate predicted binding modes	AutoDock Vina, Glide, GNINA, PoseBusters, MM/PBSA or MM/GBSA workflows, and MD packages such as GROMACS, AMBER, NAMD, or Desmond	Docking scores, binding poses, interaction patterns, pose-quality checks, rescoring results, MD stability descriptors, and free-energy estimates where applicable	Docking scores, rescoring values, and short MD stability should support prioritization. They should not replace biochemical or biophysical validation.
Translational and experimental readiness	Assess whether prioritized molecules can realistically enter follow-up studies	SwissADME, ADMETlab, retrosynthesis tools, reaction-prediction tools, purchasability searches, SureChEMBL, and patent-context search resources	ADMET risk annotations, route plausibility, building-block or purchasability evidence, patent-context novelty, and assay-readiness information	Translational readiness requires candidate-level judgment. ADMET predictions, retrosynthetic routes, and patent searches are risk annotations rather than definitive proof of developability, synthesis, or freedom to operate.

Note: The listed tools and platforms are representative examples rather than exhaustive or universally recommended options. Their suitability depends on the study purpose, input quality, validation setting, and availability of appropriate reference compounds or baselines.

Table S4. Practical reference values and interpretation limits for staged evaluation of AI-generated molecules.

Evaluation Item	Common Reference Value or Practice	What It Can Indicate	Main Interpretation Limit
Validity	Report the fraction of parsable and sanitized molecules. For 3D generators, include geometric or pose-validity checks where relevant.	Basic molecular correctness and representation-level feasibility	Validity does not imply medicinal chemistry feasibility, synthesizability, or biological relevance.
Uniqueness	Report after canonicalization and specify sample size, such as Uniqueness@1000 or Uniqueness@10000.	Exact-structure redundancy in generated outputs	High uniqueness does not guarantee scaffold diversity or useful exploration.
Exact novelty	Report against a clearly defined reference set, such as the training set or external chemical databases.	Whether generated molecules are absent from the chosen reference set	Exact novelty does not imply scaffold novelty, analogue-level novelty, patent-context novelty, or practical value.
Scaffold novelty	Report scaffold definition, such as Bemis–Murcko scaffolds, and compare with relevant reference sets.	Whether generated molecules explore new core frameworks	Scaffold novelty may reduce target relevance if not balanced with biological and medicinal chemistry constraints.
Diversity	Report fingerprint type, similarity metric, and whether diversity is calculated before or after filtering.	Breadth of chemical-space exploration	High diversity can reflect chemically scattered or target-irrelevant molecules.
Lipinski Rule of Five	MW ≤ 500, cLogP ≤ 5, HBD ≤ 5, and HBA ≤ 10 are common oral drug-likeness reference values.	First-pass risk indicator for oral absorption or permeability	These values are empirical guidelines, not absolute exclusion rules.
Veber-type descriptors	Lower rotatable bond count and lower polar surface area or hydrogen-bond count are commonly used as oral bioavailability-related references.	Potential risk related to flexibility, polarity, and oral exposure	These descriptors do not determine permeability or bioavailability in a compound-specific context.
QED	Report QED together with individual physicochemical descriptors.	Similarity to the property profile of known drugs	QED does not assess target engagement, selectivity, safety, synthesis, or clinical utility.
SA score	Use as an early estimate of synthetic tractability and report the scoring implementation.	Approximate synthetic difficulty	SA score does not provide a synthetic route, yield, cost, stereochemical control, or purchasability.
Structural alerts	Report PAINS, reactive-group, toxicophore, or internal alert filters where used.	Potential assay interference or chemical liability	Alert flags are context-dependent and should guide triage rather than blind rejection.

Activity or target-aware score	Report endpoint definition, training data, validation strategy, threshold, and applicability-domain or uncertainty information where available.	Computational evidence of target relevance	A predicted score is not experimental activity and may reflect predictor bias or reward exploitation.
Docking score	Report score distributions, ligand size such as MW or heavy atom count, reference ligands or baselines, and failed docking attempts where relevant.	Relative structure-based prioritization	Docking scores are not direct measurements of binding affinity or potency.
Pose and interaction analysis	Report pose plausibility, key interactions, steric clashes, and target-specific recognition features.	Structural compatibility with the binding site	Visually plausible poses do not establish binding or activity without further evidence.
MM/PBSA or MM/GBSA	Use mainly for relative prioritization within a filtered and structurally related set.	Approximate binding free-energy ranking	Small energy differences should not be overinterpreted because results are protocol-sensitive.
MD stability and interaction persistence	Report simulation length, force field, ligand parameters, analysis window, and contact definitions.	Pose stability and interaction persistence under the simulation protocol	Short MD stability or repeated contacts alone should not be treated as evidence of potency.
MD-based free-energy estimation	Report the free-energy method, sampling protocol, uncertainty treatment, and validation strategy.	Affinity-oriented prioritization for a limited number of compounds	Calculated free energies depend on system preparation, sampling, force field, and validation quality.
ADMET predictions	Use as endpoint-specific risk annotations, such as solubility, permeability, CYP inhibition, hERG liability, mutagenicity, or hepatotoxicity.	Early developability risks	In silico ADMET results do not prove safety, pharmacokinetic success, or clinical developability.
Patent-context novelty	Report exact matching, similarity search, and scaffold comparison against patent-derived chemical space where relevant.	Possible overlap with disclosed chemistry	Absence from patent databases does not establish freedom to operate.
Experimental readiness	Report purchasability, route plausibility, assay compatibility, and candidate-level rationale.	Whether a molecule is realistic enough for follow-up	Computational readiness is not equivalent to experimental validation.

Note: These values are practical reference points commonly used in molecular generation, cheminformatics, medicinal chemistry, and structure-based evaluation. They should be interpreted in relation to the study objective, chemical series, target context, and available validation evidence.

Table S5. Application of the staged evaluation framework to representative molecular generation examples.

Example	Study Type	Main Evidence Illustrated	Application of the Six-Stage Framework	Main Interpretation Caution
GuacaMol and MOSES	Benchmark studies	Standardized model-level evaluation using validity, uniqueness, novelty, diversity, distributional similarity, and goal-directed benchmark tasks	Mainly addresses molecular correctness, uniqueness, novelty, diversity, and benchmark-level reporting. These benchmarks provide controlled comparisons of generative models but usually do not evaluate candidate-level synthesis, target engagement, or experimental testability.	Benchmark performance should not be interpreted as evidence that generated molecules are medicinally feasible, target-relevant, or experimentally actionable.
REINVENT	Target-directed method paper	Reinforcement learning-guided molecular generation using user-defined objectives or predictive scoring functions	Illustrates target-aware optimization. It can be evaluated by molecular correctness, medicinal chemistry feasibility, novelty, reward components, target-aware scores, and post hoc orthogonal validation.	A high reward score may reflect optimization of the scoring function rather than true biological relevance. Reward independence, applicability-domain analysis, and orthogonal evaluation are needed.
GENTRL DDR1 inhibitors	Prospective discovery study	De novo generation, prioritization, synthesis, and experimental testing of DDR1 inhibitor candidates	Covers multiple stages, including molecular correctness, medicinal chemistry feasibility, novelty, target relevance, synthetic feasibility, and experimental validation. It shows how generated molecules can move from computational proposals to tested compounds.	The strength of the claim depends on the full evaluation funnel, including the number of molecules generated, filtered, synthesized, tested, and confirmed.
Rentosertib, formerly ISM001-055	Translationally advanced AI-assisted discovery case	AI-assisted identification and development of a TNIK inhibitor for idiopathic	Illustrates the translational readiness layer. It extends beyond computational prioritization and	This case should not be treated as a universal template for all molecular generation studies. It

		pulmonary fibrosis, with reported clinical evaluation	involves synthesis, preclinical characterization, safety assessment, and clinical testing.	represents a high-evidence translational example, whereas most studies support more limited claims.
Pocket2Mol	Structure-based 3D generation method	Pocket-conditioned generation of molecules under spatial and geometric constraints from protein binding sites	Emphasizes three-dimensional validity, pocket compatibility, pose plausibility, drug-likeness, synthetic accessibility, and structure-based prioritization. It is particularly relevant to the structure-based plausibility stage.	Structure-based scores and generated poses should not be overinterpreted without geometric checks, pose inspection, realistic baselines, and, where appropriate, rescoring or molecular simulation.
